# Developing a comprehensive structured program for managing gestational diabetes mellitus and preventing type 2 diabetes mellitus in Chinese women: a multi-method study

**DOI:** 10.3389/fendo.2025.1627702

**Published:** 2025-08-01

**Authors:** Jing Huang, Tianxue Long, Hua Li, Hangyu Cui, Dan Zhang, Yi Wu, Yiyun Zhang, Xinying Sun, Yan Zhao, Yuan Wei, Mingzi Li

**Affiliations:** ^1^ School of Nursing, Peking University, Beijing, China; ^2^ Department of Social Medicine and Health Education, School of Public Health, Peking University, Beijing, China; ^3^ Department of Obstetrics and Gynecology, Peking University Third Hospital, Beijing, China

**Keywords:** gestational diabetes mellitus, type 2 diabetes mellitus, patient education, adherence, motivation

## Abstract

**Background:**

Gestational diabetes mellitus (GDM) affects pregnancy outcomes and increases the risk of type 2 diabetes mellitus (T2DM) postpartum. Traditional health education interventions have shown positive effects, but adherence to lifestyle management and motivation for continued postpartum care still require improvement. This study aimed to develop a comprehensive structured program for Chinese women with GDM to improve adherence to lifestyle management and motivation for postpartum care, thereby reducing future T2DM risk.

**Methods:**

The development of this program was divided into five steps: (1) Analysis: a summary of evidence and the analysis of needs, population characteristics, teaching content, and teaching environment; (2) Design: the design of learning objectives, teaching logic, and teaching strategies; (3) Development: the creation and development of teaching materials; (4) Implementation: the evaluation of scientific validity and rationality through an expert meeting and trial sessions; (5) Evaluation: formative and summative assessments. Finally, a pilot test was conducted to evaluate its feasibility and acceptability.

**Results:**

Learning objectives were set in the cognitive, affective, and psychomotor domains, with a total of 189 items. The program consisted of five sessions, including: understanding GDM, nutrition and physical activity guidelines, expecting moms—are you ready, postpartum weight management, and reinforcing healthy behaviors. In addition, there was a session specifically for women starting insulin treatment, with the theme: timely insulin injection. Teaching materials included lesson plans, teaching posters, food cards, and a mother’s handbook, etc., totaling 11 items. The pilot study included eight women with GDM, all of whom expressed a positive acceptance of the program.

**Conclusions:**

The feasibility and acceptability of the program were confirmed, and a final version was developed. It features clear objectives, detailed teaching content, engaging teaching materials, and standardized implementation processes, all of which are of significant importance for both short- and long-term management of GDM.

## 1 Introduction

Gestational diabetes mellitus (GDM) is defined by elevated blood glucose levels that first occur during pregnancy but do not meet the criteria for overt diabetes (1). In China, the prevalence of GDM increased from 4% in 2010 to 21% in 2020, and this trend is likely to persist due to economic development, lifestyle changes, and adjustments in fertility policies (2). GDM is not only associated with obstetric and neonatal complications but also serves as a significant risk factor for type 2 diabetes mellitus (T2DM) in affected women (3, 4). Therefore, effective management of GDM is essential for reducing both short- and long-term health risks. Lifestyle management is the cornerstone of GDM treatment (5). However, in many Chinese hospitals, GDM education is primarily delivered verbally, with its quality varying due to differences in healthcare providers’ knowledge. This approach lacks evidence-based, personalized, and systematic management. Since 2011, some hospitals in China have established one-day GDM clinics, which have shown positive effects on pregnancy outcomes (6, 7). However, the one-day clinic model does not sufficiently address long-term outcomes for women with GDM, highlighting the need for a continuous, integrated GDM program that covers both pregnancy and postpartum health risks.

Face-to-face, intensive, individualized or group-based lifestyle interventions are the most common and effective forms of GDM management (8, 9). While these programs produce positive short-term outcomes, their high cost and intensity pose challenges to long-term adherence after childbirth (10). Qualitative studies suggest that concerns about fetal health and the desire to avoid hypoglycemic medications often motivate lifestyle changes during pregnancy (11). However, these motivations often diminish postpartum, making it difficult to maintain a healthy lifestyle (12). Thus, future research should focus on behavioral theories that enhance motivation and adherence to lifestyle management during both pregnancy and postpartum, thereby improving long-term intervention effectiveness. Nudge refers to strategies such as designing choice frameworks and introducing subtle environmental cues to promote predictable behavioral changes while preserving individual autonomy (13). Nudge-based interventions have demonstrated positive outcomes in promoting healthy behavior (14, 15). Therefore, nudge strategies may effectively encourage women with GDM to adopt and maintain healthy behaviors and improve adherence to lifestyle management during pregnancy and postpartum. The Attention, Relevance, Confidence, and Satisfaction (ARCS) motivation model aims to stimulate, sustain, and reinforce behavioral motivation through the design of the teaching process (16). It has been widely applied in educational settings and has achieved significant results in enhancing learner motivation (17, 18). Hence, motivation-enhancing strategies based on this model may be effective in improving women’s motivation to adopt healthy behaviors.

A structured treatment and education program (STEP) is a patient education program that designs relevant content and procedures based on evidence, theory, and patient needs and organizes teaching activities and materials systematically using pedagogical principles (19). The program has clear learning objectives, standardized teaching methods, and customized teaching materials. It increases patient knowledge, skills, and motivation, improves health outcomes, and has been proven to be highly cost-effective (20, 21). Therefore, STEPs are evidence-based, systematic, personalized, and effective, showing great promise in addressing GDM management issues in China.

Therefore, this study aimed to develop an evidence-based, theory-driven STEP to address the existing gaps in GDM management in China. Through comprehensive care during pregnancy and postpartum, this project aims to support women with GDM in developing sustainable, healthy habits, thereby reducing their future metabolic risks. This study may also offer valuable insights to countries and regions facing similar challenges, contributing to the overall improvement of women’s health and well-being.

## 2 Materials and methods

### 2.1 Principles of development

**Figure 1:**
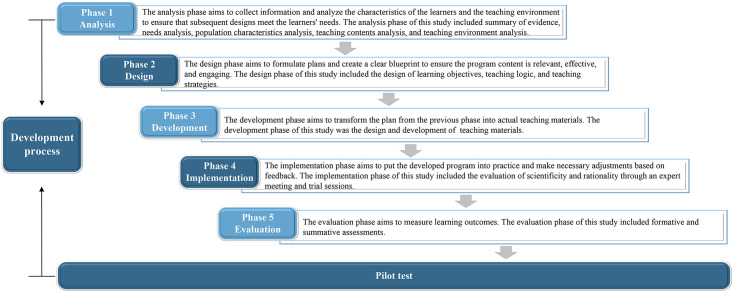
The flow diagram of the development process.

This study followed the Analysis, Design, Development, Implementation, and Evaluation (ADDIE) model for program development (22). The flow diagram of the development process is shown in **Figure 1**. The different methods of data collection and analysis used at different phases are summarized in **Table 1**. In addition, we also followed the Template for Intervention Description and Replication checklist and guide during the development process to ensure a complete description of the intervention (23).

**Table 1:** Data collection methods.

Phase	Variable	Data collection method	Informant	Data analysis method
Analysis	Evidence	Systematic review	Literature	Qualitative integration
	Needs	Systematic review	Review	JBI convergent integrated approach
		Semi-structured interview	Women with GDM	Content analysis
	Population characteristics	Literature review	Literature	Qualitative integration
	Teaching environment	Observe on the spot	Doctors, nurses, women with GDM, and the medical environment	NA
Design	Learning objectives, teaching logic, and teaching strategies	Brainstorming method	The results of the needs analysis, population characteristics analysis, teaching environment analysis, as well as the teaching content, theories, and guidelines.	NA
Implementation	The scientificity and rationality of the program	Expert meeting	Experts	NA
		Pilot sessions	Members of the research group	NA
Pilot test	The feasibility and acceptability of the program	Pilot test	Women with GDM	NA

GDM, gestational diabetes mellitus; NA, not applicable; JBI, Joanna Briggs Institute.

### 2.2 Development process

#### 2.2.1 Phase 1: analysis

The analysis phase comprised five components: evidence summary, needs analysis, population characteristics analysis, teaching content analysis, and teaching environment analysis.

##### 2.2.1.1 Summary of evidence

In April 2023, we conducted a systematic search across 9 databases and 18 websites to identify clinical decisions, guidelines, recommended practices, evidence summaries, expert consensus, and systematic reviews related to lifestyle management for women with GDM. The detailed methodology is described in our previously published study (5).

##### 2.2.1.2 Needs analysis based on a systematic review

We conducted a systematic review of PubMed, EMBASE, PsycINFO, CINAHL, and the Cochrane Central Register of Controlled Trials to collect studies related to the needs and preferences of women with GDM. The search period was from the establishment of each database up to June 2023. Relevant references from these studies were also traced to gather additional related literature. Detailed search strategies are shown in **Supplementary Table 1**.

During the systematic review, two researchers (Jing Huang and Hua Li) independently screened the literature, assessed quality, and extracted data. Discrepancies were resolved through discussion with the third researcher (Yi Wu) until a consensus was reached. Inclusion criteria were based on the PICo framework: ① participants: women with GDM or a history of GDM; ② phenomena of interest: health education needs and preferences, with no restrictions on the literature type (qualitative, quantitative, or mixed-methods studies); ③ context: any setting (hospital, community, or home). Studies that were not available in full text, duplicates, or not published in English or Chinese were excluded.

The Joanna Briggs Institute (JBI) Critical Appraisal Checklist for Qualitative Research was used to assess the quality of qualitative and mixed-methods studies’ qualitative components, while the JBI Critical Appraisal Instrument for Studies Reporting Prevalence Data was used for the quality of quantitative and mixed-methods studies’ quantitative components (24). Basic information and content on health education needs and preferences were extracted. Data were synthesized using the convergent integrated approach (25).

##### 2.2.1.3 Needs analysis based on semi-structured interviews

Based on the philosophical foundation of naturalistic inquiry and the methodology of descriptive qualitative research, semi-structured interviews were conducted to confirm and expand the findings from the systematic review. Purposive sampling was used to identify eligible women to interview at the Outpatient Department of Peking University Third Hospital from September to October 2023. The inclusion criteria were: ① diagnosis of GDM; ② age ≥ 18 years; and ③ voluntary participation and informed consent. The exclusion criteria were: ① history of psychiatric disorders; ② cognitive, visual, or hearing impairments; and ③ severe organ dysfunction or physical disabilities. The interview outlines were: ① after being diagnosed with GDM, what kind of support do you feel you need? ② what are your preferences regarding the format and setting of health education? Pre-interviews with two women with GDM were conducted to assess the feasibility of the interview outline.

Basic information of the participants was collected through questionnaires. The researcher (Jing Huang) conducted the semi-structured interviews, and another researcher (Hua Li) audio-recorded the interviews with consent and took written notes. Interviews were conducted in a quiet patient education room and lasted 5–20 minutes each. Recordings were transcribed into written form within 24 hours and analyzed using NVivo 12.0 software. The content analysis method was used for data analysis (26).

##### 2.2.1.4 The analysis of population characteristics and teaching contents

The population characteristics of women with GDM were analyzed through an extensive literature search in PubMed. During the literature search, we focused on studies that reported on sociodemographic characteristics, the current situation of self-management, and levels of social support among women with GDM, in order to gain a comprehensive understanding of the characteristics of this population. Then, the teaching contents were determined based on the results of needs analysis and population characteristics analysis.

##### 2.2.1.5 The analysis of teaching environment

We conducted on-site visits to the obstetrics outpatient clinics and nutrition outpatient clinics of four tertiary hospitals in Beijing, China, to analyze the characteristics of the teaching environment by observing the current status of GDM management.

#### 2.2.2 Phase 2: design

The design phase focused on developing a detailed teaching plan using the brainstorming method, including three key components: learning objectives, teaching logic, and teaching strategies. This was based on the guidelines, the results of needs analysis, population characteristics analysis and teaching environment analysis, teaching contents, motivation-enhancing and nudging strategies, and Knowles’ adult learning theory.

One week before the brainstorming meeting, the researcher (Jing Huang) provided the group members with printed materials related to the meeting, so that participants could familiarize themselves with the purpose and content of the meeting in advance. At the meeting, the researcher (Jing Huang) first introduced the goals of the meeting, including:

1. Identify teaching objectives: ① based on the Chinese Guideline of Diagnosis and Treatment of Hyperglycemia in Pregnancy, determine the overall goals of the program (27, 28); ② based on the analysis results of the needs, population characteristics, teaching environment, and teaching contents, using Bloom’s Taxonomy, identify the specific learning objectives of the program from the cognitive, affective, and psychomotor domains (29).
2. Identify teaching logic: considering the distinct psychological and physiological characteristics of women with GDM during pregnancy and postpartum, clarify the teaching logic by thoroughly understanding and analyzing the findings from the needs analysis and the analysis of population characteristics.
3. Identify teaching strategies: ① at the macro level, in order to improve the adherence and motivation among women with GDM during pregnancy and postpartum in managing their lifestyle, determine nudge strategies that contribute to lifestyle management adherence and promote the achievement of the learning objectives based on the Ten Important Nudges, the TIPPME (Typology of Interventions in Proximal Physical Micro-Environments), and the MINDSPACE (Messenger, Incentives, Norms, Defaults, Salience, Priming, Affect, Commitment, and Ego) frameworks (30–32), and identify motivation-enhancing strategies that contribute to strengthen both learning and lifestyle management motivation based on the attention, relevance, confidence, and satisfaction strategies in the ARCS motivation model; ② determine the teaching strategies at the micro level based on Knowles’ theory of adult learning (33).

Then, the researcher emphasized that group members should actively participate in discussions, with no right or wrong answers, and no arbitrary criticism or interruption of others’ speech. The researcher was also responsible for controlling the meeting time and progress. Another researcher (Tianxue Long) audio-recorded the meeting with consent and took written notes. Before concluding the discussion on each issue, the researcher (Jing Huang) summarized the discussion points and asked if there were any additions. If no new content emerged, the meeting moved to the next issue until all topics were covered. Finally, the facilitator and the recorder summarized and organized the content determined during the meeting, forming the program’s learning objectives, teaching logic, and teaching strategies.

#### 2.2.3 Phase 3: development

Based on the population characteristics, needs, teaching environment, teaching contents, learning objectives, teaching logic, teaching strategies, evidence summaries, relevant authoritative literature and books, and the STEP previously developed by our research team for patients with T2DM, the first draft of the teaching materials was designed and developed in collaboration with a graphic design professional (34).

#### 2.2.4 Phase 4: implementation

##### 2.2.4.1 Expert meeting

After the first draft was constructed, an expert meeting was conducted to assess the scientificity (adherence to guidelines and clinical practices) and rationality (alignment with typical medical visits and follow-up routines for women with GDM) of the program. Expert selection criteria included: ① a Master’s degree or higher; ② an intermediate or senior professional title; and ③ voluntary participation. Experts’ basic information was collected through information forms. Before the meeting started, each expert was provided with a printed structured checklist and a tablet loaded with all the teaching materials for evaluation and review during the meeting. During the expert meeting, the researcher (Jing Huang) introduced the program’s development process and content to the experts. Then, the experts were free to review the materials on the tablet, and the researcher invited them to provide suggestions based on the structured checklist. The suggestion was adopted when the experts reached a consensus on it, and the meeting ended when no new suggestions were made. To ensure transparency and accuracy, another researcher (Tianxue Long) audio-recorded the meeting with consent and took written notes. Within 24 hours, the suggestions were summarized into a written document. Based on the feedback from the expert meeting and internal discussions within the research team, revisions were made, resulting in the development of a second version.

##### 2.2.4.2 Trial session

Based on the second version, trial sessions were conducted. Research team members role-played women with GDM and interacted with the educator (Jing Huang). The basic information of the research team members was collected through a questionnaire. After each trial session, suggestions from the team were collected, and any suggestion that reached a consensus was adopted. Another researcher (Tianxue Long) audio-recorded the discussion with consent and took written notes. The recordings were summarized and organized into a written document within 24 hours. The program was revised based on the feedback, followed by another trial session, continuing this cycle until no new suggestions emerged. After all trial sessions and revisions, the third version was finalized.

#### 2.2.5 Phase 5: evaluation

The evaluation of the program consisted of both formative and summative assessments. Formative assessment was conducted throughout the program, focusing on the use of teaching materials, classroom interactions, and completion of assignments. Summative assessment was performed at the end of the program, evaluating cognitive, affective, and psychomotor outcomes. In addition, health changes in women with GDM were assessed using objective indicators.

### 2.3 Pilot test

We conducted a pilot test of the third version in the obstetric outpatient department of a tertiary hospital in Beijing to explore its feasibility (whether the program could be successfully implemented in the clinic) and acceptability (attitudes and responses of women with GDM to the program). Inclusion criteria were: ① diagnosis of GDM according to the Chinese guideline for the diagnosis and management of hyperglycemia in pregnancy (2022) (28); ② age ≥ 18 years; ③ singleton intrauterine pregnancy; ④ ability to understand Mandarin; and ⑤ voluntary participation. Exclusion criteria were: ① history of mental illness; ② cognitive, visual, or hearing impairment; ③ serious organ damage or physical disability; ④ abnormal pregnancy conditions such as placental abruption or placenta previa; and ⑤ concurrent participation in other studies.

Obstetric physicians recruited eligible women with GDM, one researcher (Hua Li) coordinated participant enrollment, and another researcher (Jing Huang) conducted the sessions. All sessions were held in the patient education room, with each session involving one woman. After the session, feedback was collected using the question, “What do you think of the content and format of this session?” The researcher (Hua Li) audio-recorded the feedback with consent and took written notes. The recordings were summarized and organized into a written document within 24 hours. In addition, we collected participants’ pregnancy weight gain and postpartum blood glucose through the medical record system.

### 2.4 Ethical considerations

This study was reviewed and approved by the Ethics Committees of the Peking University Health Science Center (IRB00001052-23063; July 3rd, 2023) and Peking University Third Hospital (IRB00006761-M2023463; August 14th, 2023). All women with GDM provided written informed consent, and all data were kept confidential and anonymous.

## 3 Results

### 3.1 Development process

#### 3.1.1 Phase 1 analysis

##### 3.1.1.1 The results of evidence summary

A total of 12,196 records were retrieved, 55 articles were included in the analysis, and 69 pieces of evidence were summarized. The flowchart of the literature screening, the results of the quality assessment, and the evidence extraction form can be found in our previously published article (5).

##### 3.1.1.2 The results of needs analysis

In the systematic review, a total of 379 records were initially retrieved. Ten articles were included in the data analysis, of which 5 were qualitative studies, 4 were quantitative studies, and 1 was a mixed-methods study. The flowchart of the literature screening is shown in **Supplementary Figure 1**, the results of the quality assessment of the articles are shown in **Supplementary Tables 2** and **3**, and the basic characteristics of the included studies are presented in **Supplementary Table 4**. The results of the needs and preferences in the systematic review are shown in **Supplementary Table 5**. In the interviews, we conducted interviews with 21 women with GDM, and their basic information is provided in **Supplementary Table 6**. The results of the needs and preferences in the interviews are shown in **Supplementary Table 7**.

##### 3.1.1.3 The results of the analysis of population characteristics

Based on the analysis of population characteristics, the program design should consider the cognitive abilities of different educational groups, combining both face-to-face and online education to alleviate women’s time pressures. It should also adopt effective strategies to improve adherence to lifestyle management, enhance motivation for postpartum management, strengthen social support, and include comprehensive management throughout both the prenatal and postpartum periods. The detailed results are shown in **Table 2**.

**Table 2:** The results of the analysis of population characteristics.

Population characteristics	Results of analysis
Age	The majority of women with GDM in China are young, with over 90% being under 35 years old, and most have good listening, speaking, reading, and writing abilities (2). Therefore, developing printed teaching materials is acceptable for this group. However, this young group is also at a critical stage where family and social responsibilities overlap, with a higher proportion of women continuing to work during pregnancy and needing to return to work after childbirth, which adds pressure on them to balance family and social responsibilities (35). Therefore, the program design needs to take their time pressure into account and adopt a combination of face-to-face and online health education to better meet their needs.
Level of education	There is a significant disparity in the educational levels of women with GDM in China (36). Therefore, the program design should fully consider the cognitive abilities of different educational groups, ensuring that the content remains professional while using clear and simple language to ensure that all audiences can understand and absorb the relevant knowledge.
Current status of self-management	The main motivations for women with GDM to engage in self-management are concerns about fetal health and avoiding the use of blood sugar-lowering medications (11). However, despite strong self-management motivation during pregnancy, there is still a problem with poor lifestyle adherence during pregnancy, and the issue becomes even more serious after childbirth (37, 38). Therefore, the program design should adopt effective strategies to improve lifestyle adherence among women with GDM during both pregnancy and postpartum. In addition, as motivation decreases after childbirth, women with GDM often find it difficult to maintain lifestyle management postpartum (12). Thus, the program should also focus on enhancing the motivation for continued self-management among women with GDM after childbirth.
Level of social support	The level of social support for women with GDM in China is moderately high, but there is still room for improvement (35). Given that good social support plays a positive role in promoting lifestyle management for women with GDM, the program should focus on enhancing their social support levels.
Challenges related to the healthcare system	Women with GDM complete postpartum check-ups within 4–12 weeks in obstetrics, but the current healthcare system in China lacks a coordinated model between obstetrics and endocrinology for GDM management and T2DM prevention (27). This may lead to women with GDM neglecting postpartum lifestyle management and regular blood sugar screening (39). Therefore, the program should include both the prenatal and postpartum stages, and the design should focus on enhancing self-management adherence and reinforcing postpartum self-management motivation.

GDM, gestational diabetes mellitus; T2DM, type 2 diabetes mellitus.

##### 3.1.1.4 The results of the analysis of teaching content

Based on the population characteristics and needs analysis of women with GDM, the teaching content was determined, as shown in **Table 3**.

**Table 3:** Teaching content.

Category	Teaching content
Basic physiological knowledge	Definition of blood glucose, insulin’s role in lowering blood glucose, and the characteristics of glucose metabolism during pregnancy.
GDM-related knowledge	Definition, diagnostic criteria, pathogenesis, risk factors, short- and long-term effects on mothers and offspring, key factors for its occurrence and control, postpartum blood glucose changes, identification and prevention of neonatal hypoglycemia, risk of T2DM, the relationship between postpartum weight and T2DM, and importance of continued lifestyle management after childbirth.
Treatment of GDM	(1) Dietary management: methods for food selection, individualized dietary assessment and guidance, healthy cooking methods, meal distribution methods, and understanding of sugar-free foods.
	(2) Physical activity: the importance of physical activity during pregnancy, contraindications for exercise during pregnancy, and physical activity guidelines during pregnancy.
	(3) Medication treatment: indications for adding insulin, insulin injection and storage methods, commonly used insulins during pregnancy, the safety of insulin use during pregnancy, and the use of metformin.
Self-monitoring during pregnancy	Blood glucose monitoring methods, use of blood glucose meters, recognition and management of hypoglycemia, weight monitoring methods, and urine ketone monitoring methods.
Management goals	Blood glucose management goals during pregnancy, weight management goals during pregnancy, and postpartum weight management goals.
Breastfeeding	Knowledge of breastfeeding, the relationship between breastfeeding and T2DM, insulin use and breastfeeding.
T2DM prevention guidance	Dietary management, physical activity, weight management, and breastfeeding.
Postpartum follow-up	The importance of postpartum blood glucose recheck, postpartum follow-up and monitoring.
Emotional and sleep adjustment	Meditation.

GDM, gestational diabetes mellitus; T2DM, type 2 diabetes mellitus.

##### 3.1.1.5 The results of the analysis of teaching environment

According to the requirements of basic public health services in China, pregnant women are required to visit hospitals for registration and management, and all levels of hospitals provide registration and management services for pregnant women. The prenatal examination and postpartum follow-up for women with GDM are completed at outpatient clinics, and hospitalization is not needed if no abnormalities are found. Lifestyle management such as dietary adjustments, physical activity, and weight control is generally carried out by the women themselves or with family support at home. Therefore, program design should consider the availability of teaching resources at different hospital levels and the practicality of the knowledge and skills for home environment application.

#### 3.1.2 Phase 2 design

In the design phase, 7 members of the research team participated in a brainstorming meeting. The basic information of the group members is shown in **Supplementary Table 8**. During the brainstorming session, the teaching logic was clarified, learning objectives were established, and teaching strategies were determined. The analysis approach and results for these steps are as follows.

##### 3.1.2.1 The results of the design of teaching logic

**Figure 2:**
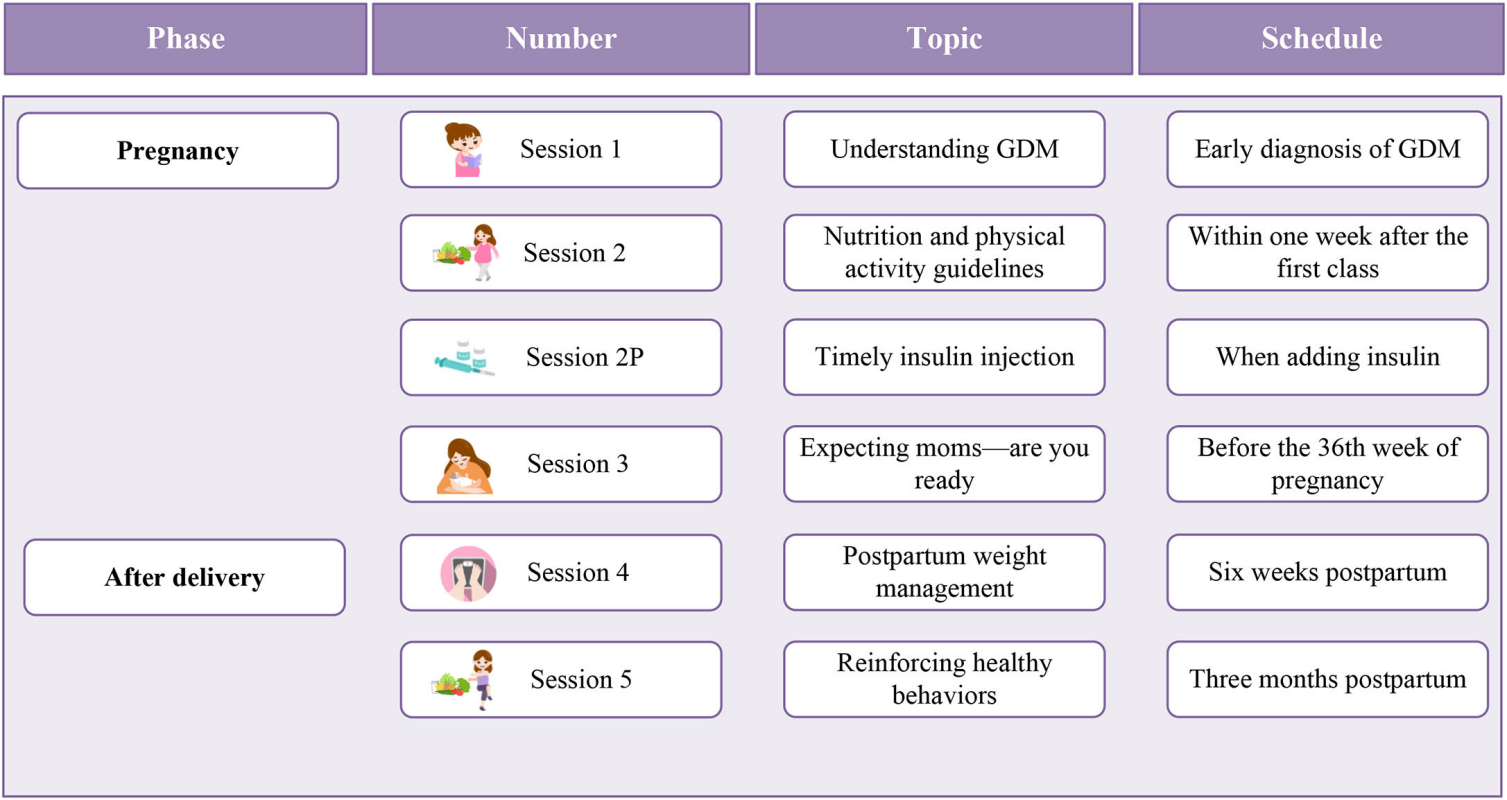
The results of the design of teaching logic. GDM, Gestational diabetes mellitus; P, personalized.

Based on the brainstorming meeting, through the analysis of the population characteristics and needs of women with GDM, we found that most lack knowledge and skills for self-management during pregnancy. They are concerned about the potential harm of hyperglycemia to the fetus and urgently need to acquire self-management knowledge and skills. In the postpartum period, the blood glucose levels of most women return to normal due to hormonal changes, leading them to overlook the long-term impacts of GDM. Further, the current clinical management model in China provides insufficient postpartum support, which may exacerbate unhealthy lifestyle behaviors. Therefore, prenatal sessions should focus on helping women quickly acquire self-management knowledge and skills while increasing their awareness of long-term health risks. Postpartum sessions should emphasize continued self-management after childbirth and reinforce the learned knowledge and skills. Based on the logic, we designed five sessions, as detailed in **Figure 2**. Among them, the personalized session was developed specifically for women who start insulin treatment.

##### 3.1.2.2 The results of the design of learning objectives

According to the brainstorming meeting, the overall goal of the program was to assist women with GDM in acquiring the knowledge and skills necessary for self-management, improving self-management behaviors and metabolic control, enhancing pregnancy outcomes, and reducing the risk of developing T2DM. The specific learning objectives were based on the characteristics and needs of the GDM population as well as the teaching content and in accordance with Bloom’s Taxonomy. The cognitive domain included 103 objectives, the psychomotor domain included 66 objectives, and the affective domain included 20 objectives, as detailed in **Supplementary Table 9**.

##### 3.1.2.3 The results of the design of teaching strategies

**Figure 3:**
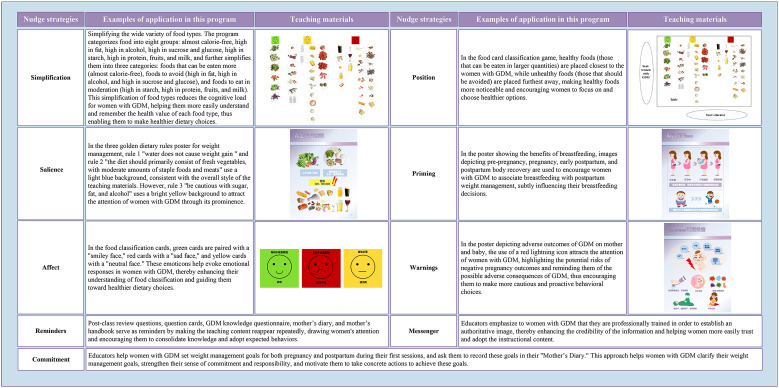
Examples of the application of nudge strategies in the program. GDM, Gestational diabetes mellitus.

**Figure 4:**
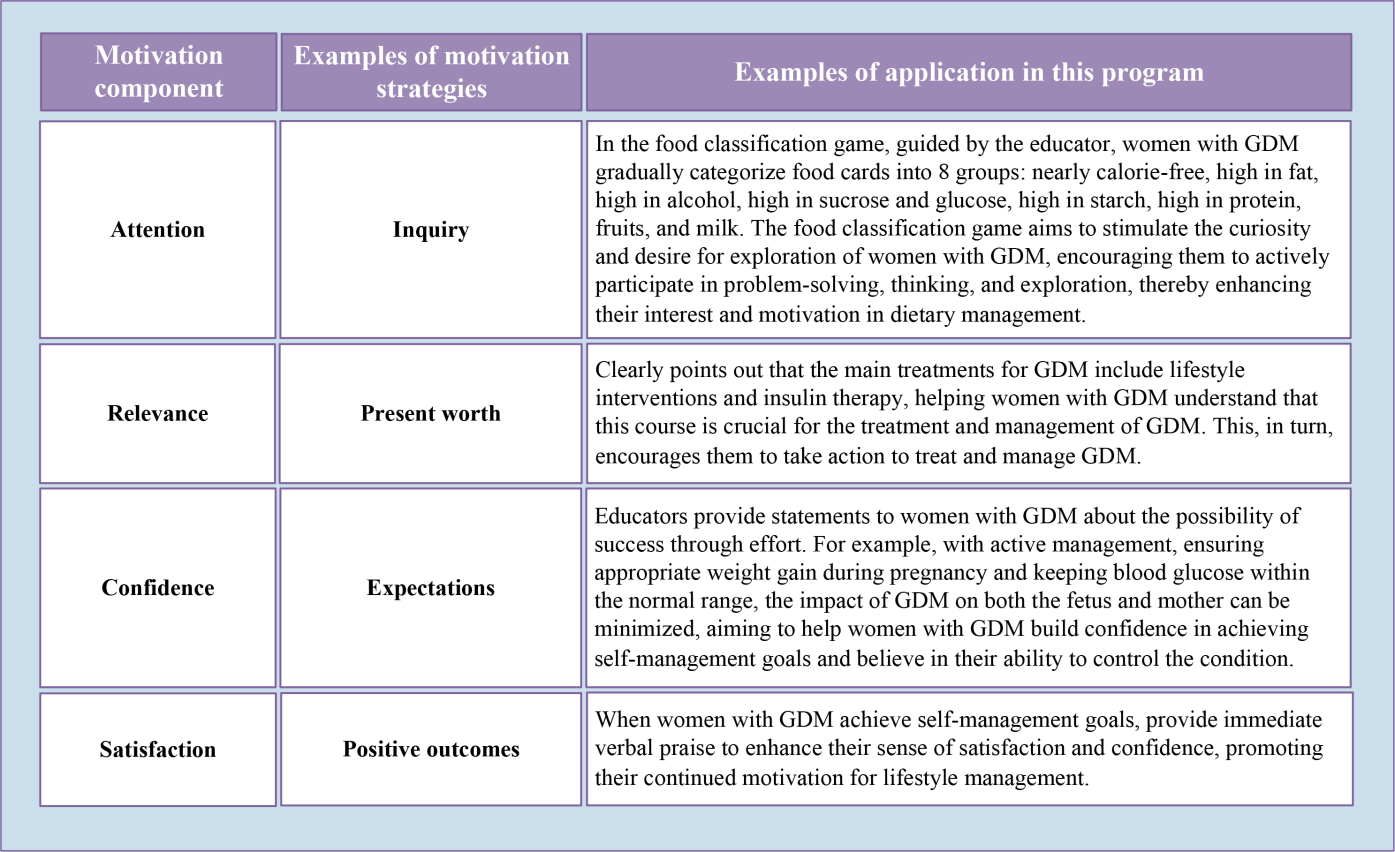
Examples of the application of motivation-enhancing strategies in the program. GDM, Gestational diabetes mellitus.

Based on the brainstorming meeting, at the macro level, the nudge strategies applied in this program included messenger, simplification, salience, affect, position, priming, commitment, warnings, and reminders, while the motivational enhancement strategies included attention, relevance, confidence, and satisfaction. **Figures 3** and **4** provide examples of these strategies, detailed in **Supplementary Tables 10** and **11**.

At the micro level, this program combined group-based teaching with individualized guidance. Group education encourages active participation and discussion, while individualized guidance addresses specific needs. To ensure full participation in discussions and to raise personalized questions, the group size was limited to 10 participants. The program utilized various teaching strategies, including lectures, questions and answers, discussions, demonstrations, games, exercises, and assignments, promoting a problem-centered learning process. One to two family members were recommended to participate in each session. Each session began with experience sharing and a knowledge review, and ended with assignments and distribution of educational materials aligned with the teaching content. The assignments included self-management tasks, such as self-monitoring of blood glucose, urine ketones, weight, and diet. Sessions were primarily in-person, but Session 5 included a PowerPoint for online participation due to postpartum time pressures. The program was implemented by trained doctors or education nurses.

#### 3.1.3 Phase 3 development

**Figure 5:**
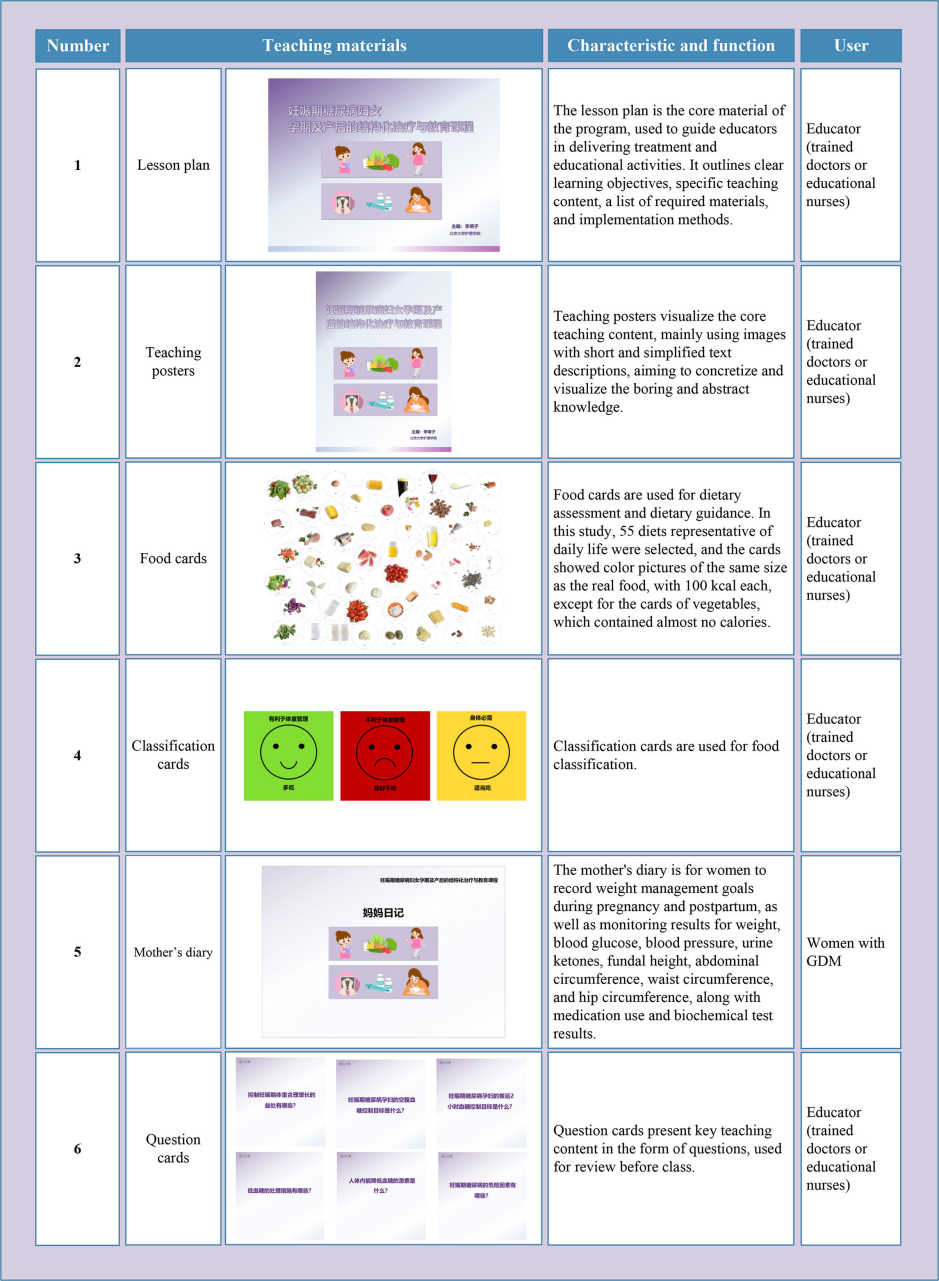
The teaching materials developed in this study (A). GDM, Gestational diabetes mellitus.

**Figure 6:**
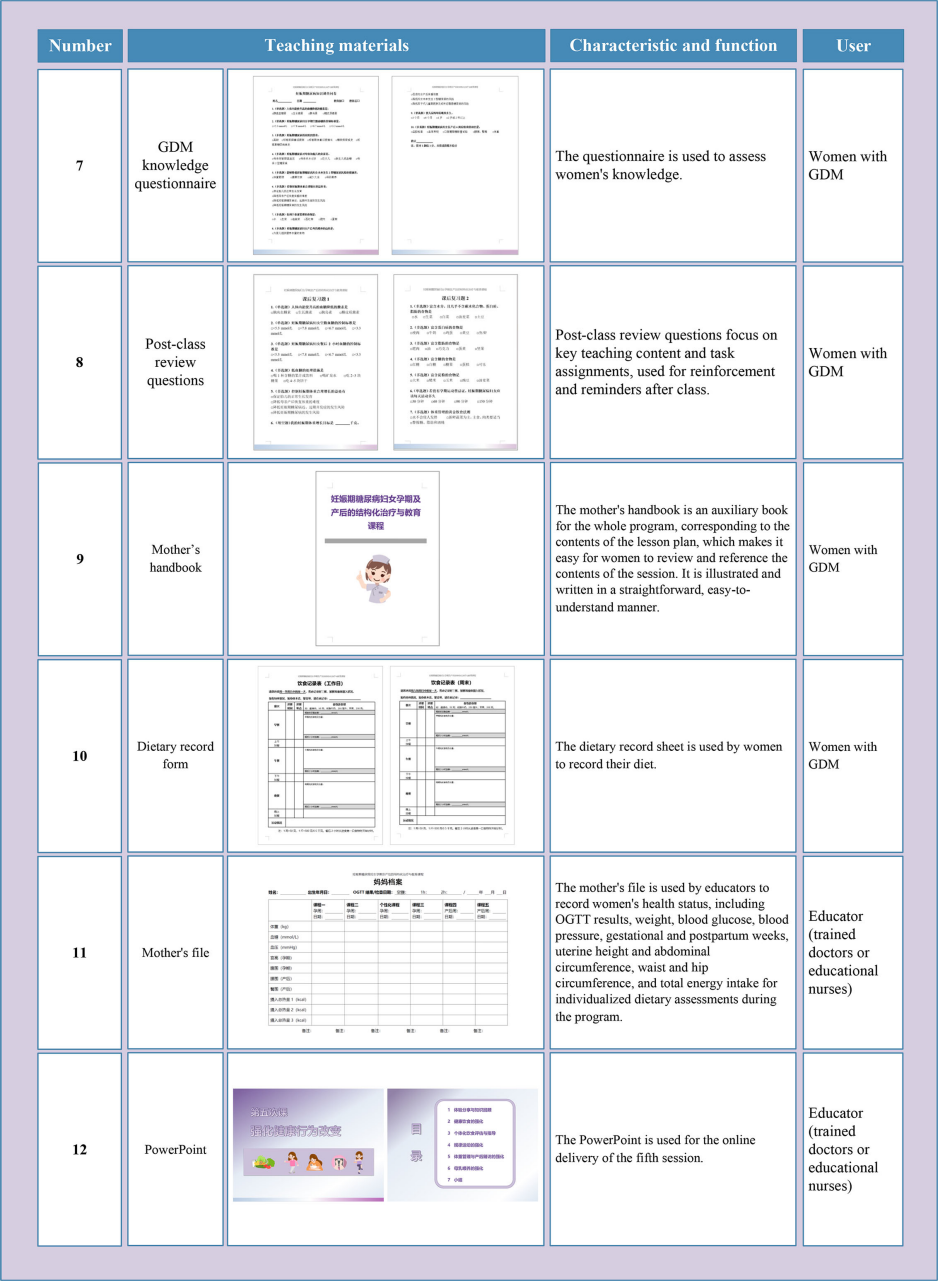
The teaching materials developed in this study **(B)**. GDM, Gestational diabetes mellitus; OGTT, oral glucose tolerance test.

The developed teaching materials were in paper format, portable, and can be used in any setting. All teaching materials were printed in color, with vivid and engaging content that is easy for women with GDM from various educational backgrounds to understand and accept. Specific items for teaching materials are shown in **Figures 5** and **6**.

#### 3.1.4 Phase 4 implementation

##### 3.1.4.1 Expert meeting

After the first draft was formed, this study invited 6 experts from the fields of obstetrics, nutrition, public health, chronic disease management, and psychology to participate in an expert meeting. The basic characteristics of the experts are shown in **Supplementary Table 12**. The meeting lasted for one hour, and the summary of the experts’ suggestions is shown in **Supplementary Table 13**.

##### 3.1.4.2 Trial session

After revising the teaching materials based on the experts’ opinions, 7 members of the research team participated in the trial sessions, and the basic information of the group members is shown in **Supplementary Table 8**. During the trial, one researcher (Jing Huang) served as the educator, while the remaining 6 team members played the role of women with GDM and interacted with the educator. After each trial, the program was improved based on feedback from the team members and retaught until no new suggestions were made. The trial process and feedback are provided in **Supplementary Table 14**.

#### 3.1.5 Phase 5 evaluation

Formative assessment included aspects such as the use of the mother’s diary, responses to questions, and completion of post-class review exercises. Summative assessment involved evaluation in the cognitive domain using the GDM knowledge questionnaire, assessment in the psychomotor skills domain through food frequency questionnaires and pregnancy physical activity questionnaire, and evaluation in the affective domain using diabetes management self-efficacy scale, regulation of eating behaviors scale, and perceived social support scales. Moreover, health changes were assessed through objective indicators such as weight, blood glucose, blood lipids, waist circumference, hip circumference, and glycated hemoglobin.

### 3.2 Pilot test

In March 2024, 8 women with GDM participated in the pilot study, and the basic information of the participants is provided in **Supplementary Table 15**. For women shortly after the diagnosis of GDM (24–30 weeks), we provided sessions 1 and 2; for women using insulin, we offered session 2P (personalized); for women in the later stages of pregnancy (32 to 36 weeks), we provided session 3; and for postpartum women attending follow-up, we provided session 4. Since the purpose of session 5 is reinforcement and its content was learned previously, it was not delivered to the participants.

Among the 6 participants who attended the pregnancy session, 4 women had appropriate weight gain during pregnancy, while 2 women gained excessive weight. Among the 8 participants, 7 women underwent an OGTT within 4–12 weeks postpartum. The pregnancy weight gain and postpartum blood glucose of participants are shown in **Supplementary Table 15**. The interview found that women with GDM highly endorsed the program and believed it contributed to improving their disease-related knowledge and their self-management knowledge and skills. No further suggestions or revisions were provided by the participants, and the feedback is shown in **Supplementary Table 16**. At this point, the final version of the program is built. The description and design of the program are shown in **Table 4**, the printed version is presented in **Supplementary Figure 2**.

**Table 4:** Description and design of the program.

Sessions	Contents/chapters	Course time and duration	Format	Teaching materials
Session 1: understanding gestational diabetes mellitus	(1) Program introduction and overview (2); share your thoughts (3); what is GDM (4); the glucose-lowering effect of insulin (5); characteristics of glucose metabolism during pregnancy (6); risk factors for GDM (7); adverse effects on the mother and fetus (8); blood glucose control targets for GDM (9); symptoms and management of hypoglycemia (10); monitoring and recording of blood glucose (11); monitoring and recording of urine ketones (12); weight monitoring and management goals (13); self-psychological adjustment-meditation (14); summary.	Course time: shortly after GDM diagnosis; course duration: 60–90 minutes.	Group-based, face-to-face education. It is recommended to have 4–10 women with GDM per group, with each woman bringing 1–2 family members. Educator: trained doctors or educational nurses.	Table, chair, lesson plan, teaching poster, mother’s diary, pen, GDM knowledge questionnaire, mother’s handbook, post-class review questions, dietary record form, meditation audio, height and weight scale, blood pressure monitor, blood glucose meter and test strips, and urine ketone test strips.
Session 2: nutrition and physical activity guidelines	(1) Experience sharing and knowledge review (2); energy-delivering nutrients (3); calories in different types of foods (4); identifying foods with almost no calories (5); identifying foods high in fat (6); identifying alcoholic beverages and foods high in sucrose and glucose (7); golden dietary rules for weight management (8); tips for blood glucose management (9); what are sugar-free foods (10); personalized dietary assessment and guidance (11); contraindications for exercise during pregnancy (12); get moving: reduce sedentary time (13); summary.	Course time: within 1 week of the first course; course duration: 60–90 minutes.	Group-based, face-to-face education, including personalized dietary guidance. It is recommended to have 4–10 women with GDM per group, with each woman bringing 1–2 family members. Educator: trained doctors or educational nurses.	Table, chair, lesson plan, teaching poster, question cards, food cards, classification cards, post-class review questions, pen, height and weight scale, blood pressure monitor, and blood glucose meter and test strips.
Session 2P: timely insulin initiation	(1) Experience sharing and knowledge review (2); indications for starting insulin therapy (3); introduction to insulin pens and storage methods (4); insulin injection techniques (5); commonly used insulin during pregnancy and its characteristics (6); the relationship between insulin, food, and blood glucose (7); oral hypoglycemic medications (8); summary.	Course time: when adding insulin; course duration: 40–60 minutes.	Group-based, face-to-face education. It is recommended to have 4–10 women with GDM per group, with each woman bringing 1–2 family members. Educator: trained doctors or educational nurses.	Table, chair, lesson plan, teaching poster, question cards, food cards, insulin pen and cartridge, post-class review questions, pen, height and weight scale, blood pressure monitor, and blood glucose meter and test strips.
Session 3: expecting moms — are you ready?	(1) Experience sharing and knowledge review (2); postpartum blood glucose changes (3); postpartum first follow-up (4); benefits of breastfeeding (5); postpartum dietary guidance (6); guidance on puerperal activities (7); summary.	Course time: before 36 weeks of pregnancy; course duration: 40–60 minutes.	Group-based, face-to-face education. It is recommended to have 4–10 women with GDM per group, with each woman bringing 1–2 family members. Educator: trained doctors or educational nurses.	Table, chair, lesson plan, teaching poster, question cards, food cards, classification cards, dietary record form, post-class review questions, pen, height and weight scale, blood pressure monitor, and blood glucose meter and test strips.
Session 4: postpartum weight management	(1) Experience sharing and knowledge review (2); postpartum weight and type 2 diabetes risk (3); personalized dietary assessment and guidance (4); get moving: regular exercise (5); regular follow-up (6); self-psychological adjustment-meditation (7); summary.	Course time: 6 weeks after delivery; course duration: 40–60 minutes.	Group-based, face-to-face education, including personalized dietary guidance. It is recommended to have 4–10 women with GDM per group, with each woman bringing 1–2 family members. Educator: trained doctors or educational nurses.	Table, chair, lesson plan, teaching poster, question cards, food cards, classification cards, meditation audio, dietary record form, post-class review questions, pen, height and weight scale, flexible tape measure, blood pressure monitor, and blood glucose meter and test strips,
Session 5: reinforcing healthy behaviors	(1) Experience sharing and knowledge review (2); reinforcing healthy eating (3); personalized dietary assessment and guidance (4); reinforcing regular exercise (5); reinforcing weight management and postpartum follow-up (6); reinforcing breastfeeding (7); summary.	Course time: 3 months after delivery; course duration: 40–60 minutes.	Group-based, face-to-face education, or group-based online education, including face-to-face or online personalized dietary guidance, respectively. It is recommended to have 4–10 women with GDM per group, with each woman bringing 1–2 family members. Educator: trained doctors or educational nurses.	(1) Face-to-face: table, chair, lesson plan, teaching poster, question cards, food cards, classification cards, post-class review questions, GDM knowledge questionnaire, pen, height and weight scale, flexible tape measure, blood pressure monitor, and blood glucose meter and test strips (2); Online: PowerPoint, networked computer, materials for personalized dietary guidance (food cards, camera-enabled mobile phone).

GDM, gestational diabetes mellitus; P, personalized.

### 3.3 Implementation cost estimates and time requirements

The estimated implementation costs for this program include: ① wage costs (salaries for the trainers and the doctors or nurses involved in the training and implementation of the program); ② material costs (printing fees for teaching materials, estimated at 806 RMB per set for this program, with an additional 44 RMB for each additional woman with GDM participating); ③ transportation costs (postage costs for the teaching materials); ④ travel costs (travel expenses for the trainers). The time requirements for implementing this program include: ① the time for trainers and doctors or nurses to participate in training and assessment (training session time: 420 minutes; assessment session time: 420 minutes*number of doctors or nurses participating in training); ② the time for doctors or nurses to officially implement the program (420 minutes for 4–10 women with GDM). Decision-makers can refer to our cost and time estimates, and based on local wages, postage, and transportation conditions, estimate the possible costs of implementing the project locally.

## 4 Discussion

This study designed and developed a STEP during pregnancy and the postpartum period for women with GDM in China, and this article outlines all stages of its development. The program consists of five educational sessions that are strategically mapped and ordered to align with and achieve the program’s objectives, including systematic teaching materials such as lesson plans that ensure the quality and standardization of the educational process, vivid and engaging teaching posters, patient handbooks that correspond to the lesson plans, food cards for 55 common foods, question cards for pre-class review, post-class review exercises, and diaries to support self-management for women. The results of the pilot test showed that the program was well accepted among women with GDM, enhanced their relevant knowledge and skills, and had a positive impact on weight management and postnatal blood glucose screening. To the best of our knowledge, this is the first comprehensive and structured program designed to manage GDM and prevent T2DM among Chinese women.

Dietary management is a crucial component of lifestyle intervention for GDM. Guidelines from multiple countries recommend that women with GDM control their total caloric intake and optimize the macronutrient balance of carbohydrates, proteins, and fats to regulate blood glucose, weight gain, and improve pregnancy outcomes (5). A review of previous studies found that dietary guidance for women with GDM primarily relies on precise energy calculations and food weighing (8, 9). This method is associated with a high cognitive load in dietary management, which can lead to negative effects such as distress, postpartum dietary indulgence, and poor adherence (40). In this program, we aim to help women make healthier dietary decisions in daily life by providing concise dietary guidance materials, easy-to-understand educational posters, and practical dietary recommendations that are easy to follow and implement. For example, this program provides dietary guidance to women with GDM based on food cards. After the educator shows all the food cards needed for the day, the women can visually understand the types and portions of food required for the day (the food size of 100 kcal displayed on the cards is equivalent to the actual size of the food). After returning home, they only need to follow the food types and portion sizes on the food cards without needing to perform additional caloric calculations or weighing. This simplified food quantification method helps reduce the cognitive load, thereby improving adherence to dietary management. In addition, the motivation-enhancing strategy not only boosts the motivation for course learning but also focuses on enhancing the motivation for continued postpartum lifestyle management, which helps promote the development of healthy dietary habits among women with GDM. Therefore, compared to traditional dietary guidance, the dietary guidance in this study integrates both nudge strategies and motivation-enhancing strategies, not only conveying dietary knowledge but also focusing on improving dietary management adherence and motivation, which will have a positive impact on the lifelong metabolic health of women with GDM.

Previous studies have identified inadequate guidance from healthcare providers as a barrier to effective self-management among women with GDM. Ge et al. conducted interviews with Chinese women with GDM and found that some perceived healthcare advice as overly basic and lacking depth (41). Similarly, Hui et al. also interviewed women with GDM in Nepal and found that some of them felt that dietary advice was too general and that they desired more guidance (42). High-quality patient education requires a systematic and reliable instructional design model. The ADDIE model is one of the most commonly used models for instructional design, which has been widely used in the development and design of curricula in many fields such as school education, medical education, and corporate training (43, 44). This program employed this model as its framework, ensuring the integrity and high quality of the development process through meticulous design and implementation across the five key phases of analysis, design, development, implementation, and evaluation. The program is implemented by trained doctors or education nurses, using standardized lesson plans, clear learning objectives, specific teaching content, and detailed teaching materials and implementation methods, which ensures the standardization of the implementation process. The lesson plans contain the key phrases of the teaching content, allowing trained doctors and nurses to deliver the program according to the prompts, which ensures the accuracy of the content and the smoothness of the teaching process to the greatest extent possible. Therefore, even in under-resourced hospitals or clinics, the program can be effectively delivered after appropriate training, based on systematic teaching materials and standardized teaching processes. In addition, with the rapid development of information technology, future postpartum support could integrate information technology-based follow-up methods (such as mobile applications or WeChat-based reminders) as a solution to improve resource efficiency and promote long-term health management and follow-up. Future studies could integrate digitalization and intelligent management into this program, thereby more effectively enhancing women’s health throughout their entire life cycle.

Based on the analysis of the needs, population Characteristics, and teaching environment, we recommend that 1~2 family members attend the classes with the woman with GDM. Women with GDM interviewed by Wah et al. reported support from husbands, parents, and in-laws, such as help preparing meals and reminders about healthy eating (45). However, Dennison et al. and Teh et al. found that some women face resistance when preparing healthy meals because family members believe that healthy eating is not in line with traditional food culture or personal preferences (40, 46). Therefore, good social support plays a positive role in lifestyle management for women with GDM, but insufficient social support is a significant barrier. The family participation model designed by this program aims to increase family members’ understanding of GDM management and promote joint participation in lifestyle management, thus creating a more supportive environment.

Currently, lifestyle intervention programs for women with GDM mainly include face-to-face individual/group health education, remote education or counseling via phone, text messages, or emails, and digital therapies based on mobile apps (10, 47, 48). Compared to these previous interventions, the program developed in this study has the following positive aspects. In terms of objectives, it not only focuses on health management during pregnancy, but also on reducing the risk of T2DM in the long term; in terms of theoretical basis, it pays more attention to the pedagogical principles of curriculum design, activation of self-management motivation, and improvement of self-management adherence; in terms of teaching logic, it pays more attention to the continuity of health management during pregnancy and postpartum; in terms of teaching strategies, it pays more attention to the active participation of women with GDM and joint participation of their families; in terms of program development, it pays more attention to building systematic and standardized teaching materials based on the needs and evidence-based evidence. The program developed in this study considered the strengths and weaknesses of previous lifestyle intervention studies for women with GDM and addressed the current lack of systematic, personalized, effective, and sustainable approaches to GDM management in China.

However, this study also has some limitations. First, in the needs analysis, this study conducted a systematic review to comprehensively determine the health education needs and preferences of women with GDM. However, the semi-structured interviews, which aimed to confirm and expand the findings from the systematic review, were conducted only at a tertiary hospital in Beijing, China. Women in hospitals of different levels and regions may differ in terms of economic status, cultural habits, and educational levels, which may lead to varying needs for them, thus affecting the representativeness of the results of needs analysis. Nonetheless, this limitation may have little impact on the results of the study, as the results of the needs analysis based on the systematic review were comprehensive. Second, the pilot test was conducted only in a tertiary hospital in Beijing, with a sample size of only 8 cases, and participants were predominantly undergraduate or above. Participants from resource-intensive tertiary hospitals may have access to more advanced and abundant medical resources, which could cause the study results to reflect feedback more from high-resource settings. Moreover, individuals with higher education levels generally have better information comprehension abilities and higher socioeconomic status, which could make them more receptive to the program and more capable of applying the knowledge they acquire. Therefore, the sample of the pilot test may not fully represent the general situation of women with GDM from hospitals of different levels and with various educational backgrounds. Despite this, when constructing this program, we considered its applicability for women with different educational backgrounds and hospitals of different levels through population characteristics analysis and teaching environment analysis, aiming to ensure that even hospitals or clinics in under-resourced areas can effectively implement the program based on systematic teaching materials and standardized teaching procedures. Furthermore, the pilot test did not conduct a quantitative analysis, and the explanatory power of the qualitative data may be limited. Therefore, to improve the generalizability of this program, future research should consider conducting multi-center trials for broader validation across different levels of hospitals and among populations with varying educational backgrounds. Of note, a multicenter randomized controlled trial for the formal implementation of this program is underway (ChiCTR2400081700). Once completed, feedback from researchers and participants will be collected, and any suggestions for program modifications will be considered.

## 5 Conclusion

This study developed an evidence−based, theory−driven STEP tailored for women with GDM in China. The program features clear learning objectives, concise and comprehensive content, clear and vivid teaching materials, and a standardized educational process, making it easy for educators to deliver and for women with GDM to understand. This design also facilitates the promotion and replication of the program, providing practical resources and tools for the health management of women with GDM during pregnancy and postpartum, and has significant potential to promote their health throughout the life cycle. The pilot test demonstrated the feasibility and acceptability of the program, making it worthy of further clinical validation and dissemination.

## Data Availability

The raw data supporting the conclusions of this article will be made available by the authors, without undue reservation.

## Supplementary material

Supplementary Figure 1 PRISMA Flow Diagram.

Supplementary Figure 2 The printed version of the program.
