# Supplementary material for: Developing a comprehensive structured program for managing gestational diabetes mellitus and preventing type 2 diabetes mellitus in Chinese women: a multi-method study
Source: Front Endocrinol (Lausanne). 2025 Aug 1;16:1627702. doi: 10.3389/fendo.2025.1627702 (PMC12353735; doi:10.3389/fendo.2025.1627702)
Supplement: Supplementary Figure 1 — PRISMA Flow Diagram. [file DataSheet1.zip › Table 8.DOCX]

**Supplementary Table 8** The basic information of the group members.

| **Number** | **Gender** | **Age** | **Educational level** | **Research field** |
| --- | --- | --- | --- | --- |
| 1 | Female | 55 | Doctoral degree | Chronic disease management |
| 2 | Female | 25 | Doctoral student | Chronic disease management |
| 3 | Female | 28 | Doctoral student | Chronic disease management |
| 4 | Female | 26 | Master's student | Chronic disease management |
| 5 | Female | 35 | Master's Student | Chronic disease management |
| 6 | Male | 26 | Master's student | Chronic disease management |
| 7 | Female | 27 | Master's student | Chronic disease management |
