# Supplementary material for: Developing a comprehensive structured program for managing gestational diabetes mellitus and preventing type 2 diabetes mellitus in Chinese women: a multi-method study
Source: Front Endocrinol (Lausanne). 2025 Aug 1;16:1627702. doi: 10.3389/fendo.2025.1627702 (PMC12353735; doi:10.3389/fendo.2025.1627702)
Supplement: Supplementary Figure 1 — PRISMA Flow Diagram. [file DataSheet1.zip › Table 9.DOCX]

**Supplementary Table** **9** Learning objectives.

| **Sessions** | **Teaching content** | **Learning objectives** |
| --- | --- | --- |
| Session 1: understanding gestational diabetes mellitus | (1) Program introduction and overview | 1. Feel welcomed and valued (A). 2. Understand that lifestyle interventions and insulin therapy are the primary therapies for GDM (C). 3. Recognize their own importance in the treatment and management of GDM (A). 4. Be motivated to actively engage in their treatment (A). 5. Establish a trusting relationship with the educators (A). 6. Master the use of the mother’s diary (P). |
|  | (2) Share your thoughts | 1. Introduce themselves (P). 2. State their thoughts, wishes, problems, or confusion (P). 3. Assess the knowledge level through a GDM knowledge questionnaire (C). |
|  | (3) What is GDM | 1. Recognize that the Oral Glucose Tolerance Test is a method for diagnosing GDM (C). 2. Understand that insulin has a glucose-lowering effect (C). 3. Understand that insulin is produced by the pancreas (C). |
|  | (4) The glucose-lowering effect of insulin | 1. Understand what each color in Chart 4 represents (C). 2. Understand that the human body is composed of countless cells (C). 3. Understand that starch is broken down into glucose molecules during digestion (C). 4. Understand that the more glucose that enters the bloodstream from the intestines, the higher the blood sugar levels (C). 5. Understand that glucose must enter the cells in order to provide energy (C). 6. Understand that insulin is required for glucose to enter the cells (C). 7. Understand how elevated blood glucose levels are reduced in individuals without diabetes (C). |
|  | (5) Characteristics of glucose metabolism during pregnancy | 1. Understand that certain substances secreted by the body during pregnancy can interfere with the normal functioning of insulin (C). 2. Understand that insulin demand increases during pregnancy, and healthy pregnant women are able to secrete enough insulin to meet this heightened demand (C). 3. Understand that women with GDM cannot maintain normal blood glucose levels due to insufficient insulin secretion (C). 4. Understand that obesity further increases the insulin demand in pregnant women (C). |
|  | (6) Risk factors for GDM | 1. Understand that the exact cause of GDM has not yet been fully determined (C). 2. Understand that pregnant women with risk factors are more likely to develop GDM (C). 3. Understand what the risk factors for GDM are (C). 4. Understand that improving and controlling modifiable risk factors is key to managing GDM (C). |
|  | (7) Adverse effects on the mother and fetus | 1. Understand the adverse effects of GDM on both the fetus and the mother (C). 2. Understand that proactive treatment and management can significantly reduce the negative impacts of GDM on both mother and child (C). 3. Recognize the importance of GDM treatment and management (A). |
|  | (8) Blood glucose control targets for GDM | 1. Be able to state the blood glucose control targets for pregnant women with GDM (C). 2. Understand that blood glucose levels below the normal range are referred to as hypoglycemia (C). 3. Understand that both excessively low and high blood glucose levels can lead to coma (C). 4. Understand that the blood glucose level of a woman with GDM should never fall below 3.3 mmol/L at any time (C). 5. Understand that hypoglycemia can occur when there is insufficient food intake, excessive physical activity, or insulin administration (C). |
|  | (9) Symptoms and management of hypoglycemia | 1. Recognize the symptoms of hypoglycemia (C). 2. Master the methods for managing hypoglycemia (P). 3. Understand that it is important to carry candy or cookies with you at all times when undergoing insulin treatment (C). |
|  | (10) Monitoring and recording of blood glucose; | 1. Monitor blood glucose correctly and record it (P). 2. Adhere to regular self-monitoring of blood glucose (P). |
|  | (11) Monitoring and recording of urine ketones | 1. Understand that positive urine ketones are associated with inadequate carbohydrate intake and diabetic ketoacidosis (C). 2. Be aware of the appropriate timing for urine ketone monitoring (C). 3. Correctly monitor urine ketones and document the results (P). |
|  | (12) Weight monitoring and management goals | 1. Understand the sources of weight gain during pregnancy (C). 2. Understand that the recommended range for weight gain during pregnancy is determined by pre-pregnancy BMI (C). 3. Understand the meaning of different BMI categories (C). 4. Master the calculation methods for BMI and ideal body weight (P). 5. Determine your pre-pregnancy BMI level (P). 6. Know weight gain goals during pregnancy(C). 7. Know whether your current weight gain is appropriate (C). 8. Master the correct use of pregnancy weight gain charts (P). 9. Understand the risks of excessive weight gain and insufficient weight gain during pregnancy (C). 10. Master the correct method of weighing (P). 11. Adhere to regular weighing (P). |
|  | (13) Self-psychological adjustment-meditation | 1. Regulate emotions through meditation (A). |
|  | (14) Summary | 1. Agree to record their diet at home (P). 2. Agree to self-monitor at home and record results (P). 3. Agree to bring the mother’s diaries to the next session (P). 4. Agree to attend the second session at the scheduled time (P). 5. Feel that they are welcomed and valued (A). |
| Session 2: nutrition and physical activity guidelines | (1) Experience sharing and knowledge review | 1. Report on their experiences (P). 2. Answer the questions on the question cards (C). |
|  | (2) Energy-delivering nutrients | 1. Know the main classifications of nutrients in food (C). 2. Know that carbohydrates, proteins, and fats provide energy to the body (C). 3. Be able to provide examples of foods rich in protein, fats, and carbohydr ates (P). 4. Know that sugars and starches are collectively referred to as carbohydrates (C). 5. Know that foods rich in carbohydrates are primarily responsible for raising blood glucose levels (C). |
|  | (3) Calories in different types of foods | 1. Know how many kilocalories are contained in each of 1 gram of water, protein, carbohydrates, ethanol (alcohol), and fat (C). |
|  | (4) Identifying foods with almost no calories | 1. Know that foods rich in water and virtually free of carbohydrates, fats, and proteins are recommended for weight control (C). 2. Select water-rich, almost calorie-free foods from food cards (P). 3. Know that foods rich in water and containing few calories are mainly fresh vegetables (C). 4. Know that potatoes, yams, taro, and lotus root do not belong to this category of almost calorie-free vegetables (C). |
|  | (5) Identifying foods high in fat | 1. Know that the leftover food cards contain 100 kcal each (C). 2. Know that fat-rich foods are not conducive to weight management (C). 3. Select fat-rich foods from the food cards (P). |
|  | (6) Identifying alcoholic beverages and foods high in sucrose and glucose | 1. Know that foods containing alcohol are not conducive to weight management (C). 2. Know that alcohol may cause fetal malformations (C). 3. Be able to identify foods containing alcohol from food cards (P). 4. Know that foods rich in sucrose and glucose are not conducive to weight management (C). 5. Be able to identify foods rich in sucrose and glucose from food cards (P). 6. Distinguish between foods high in protein and foods high in starch (P). 7. Know that foods rich in protein, foods rich in starch, fruits, and milk should be consumed in moderation (C). |
|  | (7) Golden dietary rules for weight management | 1. Distinguish between three categories of foods: ① foods that support weight management and can be consumed in larger portions; ② foods that are essential for the body and should be consumed in moderation; ③ foods that interfere with weight management and should be minimized or avoided (P). 2. Understand the three golden dietary rules for weight management (C). |
|  | (8) Tips for blood glucose management | 1. Know that smaller, more frequent meals are beneficial for glycemic control and prevention of starvation ketosis (C). 2. Mast the proper way to portion meals (P). 3. Know that dietary fiber is beneficial in controlling blood glucose and blood cholesterol (C). 4. Know that replacing fruit for fruit juice, whole grain bread for white bread, and mixed grain rice for white rice can increase dietary fiber intake (C). |
|  | (9) What are sugar-free foods | 1. Understand that artificial sweeteners contain no calories and can be used as substitutes for sucrose to enhance the sweetness of foods (C). 2. Understand that sugar alcohols contain calories, and excessive consumption may not be conducive to weight management (C). 3. Know that it is important to control the intake of sugar-free food (C). |
|  | (10) Personalized dietary assessment and guidance | 1. Know if the current diet is appropriate (C). 2. Manage diet according to dietary recommendations (P). |
|  | (11) Contraindications for exercise during pregnancy | 1. Know that exercise helps to control blood glucose and weight (C). 2. Know the contraindications to exercise during pregnancy (C). |
|  | (12) Get moving: reduce sedentary time | 1. Knows that pregnant women without contraindications to exercise during pregnancy are able to perform appropriate activities (C). 2. Knows that walking is the easiest form of activity (C). 3. Knows the indications for stopping activity during pregnancy (C). 4. Agree to choose the appropriate way of regular activity according to physical condition and personal preference (P). |
|  | (13) Summary | 1. Agreed to self-monitor at home and record results (P). 2. Agreed to bring their mother’s diary to the next session (P). 3. Agree to attend the third session at the scheduled time (P). |
| Session 2P^a^: timely insulin initiation | (1) Experience sharing and knowledge review | 1. Report on their experiences (P). 2. Answer the questions on the question cards (C). |
|  | (2) Indications for starting insulin therapy | 1. Understand that after insulin injections and blood glucose control, the risks of maternal hyperglycemia to the fetus can be reduced (C). 2. Know that insulin injections do not adversely affect the fetus (C). 3. Correctly face changes in treatment regimen (A). |
|  | (3) Introduction to insulin pens and storage methods | 1. Know that injecting insulin does not hurt (C). 2. Know how to store insulin (P). |
|  | (4) Insulin injection techniques | 1. Inject insulin correctly (P). 2. Know the appropriate injection site (C). 3. Know that the injection site should be changed daily (C). 4. Inject vertically and pinch up the skin if there is little fat tissue (P). 5. Know that needles should be disposable (C). |
|  | (5) Commonly used insulin during pregnancy and its characteristics | 1. Know the different types of insulin and their action characteristics (C). 2. Know the type of insulin they inject and its action characteristics (C). 3. Record their current insulin injection dose and the time of injection (P). |
|  | (6) The relationship between insulin, food, and blood glucose | 1. Know that hypoglycemia can occur if carbohydrates are not consumed promptly after insulin injection (C). 2. State the symptoms and management of hypoglycemia (C). 3. Know that dietary management should be continued after insulin injection (C). |
|  | (7) Oral hypoglycemic medications | 1. Know that metformin can be used to control blood glucose during pregnancy (C). 2. Know that there are no known adverse effects of metformin on the fetus (C). |
|  | (8) Summary | 1. Agree to self-monitor at home and record results (P). 2. Agree to bring their mother’s diary to the next session (P). 3. Agree to attend the third session at the scheduled time (P). |
| Session 3: expecting moms — are you ready? | (1) Experience sharing and knowledge review | 1. Report on their experiences (P). 2. Answer the questions on the question cards (C). |
|  | (2) Postpartum blood glucose changes | 1. Understand that anti-insulin substances disappear with the delivery of the placenta and that blood glucose returns to normal in most pregnant women (C). 2. Know that some women with GDM still have abnormal blood glucose after delivery (C). 3. Know that if women with abnormal blood glucose after delivery have timely lifestyle management, future T2DM can be avoided as much as possible (C). 4. Recognize the importance of continuing lifestyle management after delivery (A). |
|  | (3) Postpartum first follow-up | 1. Know the purpose of the initial postpartum review (C). 2. Know the items to be included in the initial postpartum review (C). 3. Recognize the importance of postnatal review (A). 4. Know the need for continued dietary management, regular exercise, and weight control even if the postnatal blood glucose screening result is normal (C). |
|  | (4) Benefits of breastfeeding | 1. Know the recommended duration of breastfeeding (C). 2. Know that a woman with GDM can breastfeed (C). 3. Know that breastfeeding is possible for women on insulin (C). 4. Know that breastfeeding can help prevent T2DM in women with GDM (C). 5. Recognize the symptoms of neonatal hypoglycemia (P). 6. Know how to prevent hypoglycemia in newborns (P). |
|  | (5) Postpartum dietary guidance | 1. Recognize the importance of continuing a healthy diet postpartum (A) 2. Distinguish between three categories of foods: ① foods that support weight management and can be consumed in larger portions; ② foods that are essential for the body and should be consumed in moderation; ③ foods that interfere with weight management and should be minimized or avoided (P). 3. Understand that soup contains minimal nutrients and is primarily used to supplement hydration (C). |
|  | (6) Guidance on puerperal activities | 1. Know the importance of puerperal activities (C). 2. Know the types of activities that can be performed during the puerperium (C). 3. Agree to start physical activity gradually during the puerperal period according to the physical recovery (P). |
|  | (7) Summary | 1. Agree to record their diet at home (P). 2. Agreed to self-monitor at home and record results (P). 3. Agreed to bring their mother’s diary to the next session (P). 4. Agree to attend the fourth session at the scheduled time (P). |
| Session 4: postpartum weight management | (1) Experience sharing and knowledge review | 1. Report on their experiences (P). 2. Answer the questions on the question cards (C). |
|  | (2) Postpartum weight and type 2 diabetes risk | 1. Know that they have a high risk of developing T2DM in the future (C). 2. Know that a healthy diet, regular exercise, and weight management can reduce their future risk of T2DM (C). 3. Recognize the importance of postpartum weight management (A). 4. Know their postpartum weight management goals (C). |
|  | (3) Personalized dietary assessment and guidance | 1. Distinguish between three categories of foods: ① foods that support weight management and can be consumed in larger portions; ② foods that are essential for the body and should be consumed in moderation; ③ foods that interfere with weight management and should be minimized or avoided (P). 2. Know if the current diet is appropriate (C). 3. Manage diet according to dietary recommendations (P). |
|  | (4) Get moving: regular exercise | 1. Know that exercise can promote weight regain, which is conducive to reducing the risk of T2DM and GDM recurrence in the future (C). 2. Know that regular exercise can be started gradually after puerperium if the body recovers well (C). 3. Know that exercise is very important (C). 4. Exercise consistently as much as possible (except for those who are limited in activity for physical reasons) (P). |
|  | (5) Regular follow-up | 1. Recognize the importance of regular postnatal follow-up (A). 2. Regular follow-up: if the initial postnatal blood glucose screening result is normal, then follow-up every 1-3 years thereafter; if the initial postnatal blood glucose screening result is abnormal, then management and treatment in endocrinology specialists should be carried out in a timely manner (P). |
|  | (6) Self-psychological adjustment-meditation | 1. Regulate emotions through meditation (A). |
|  | (7) Summary | 1. Agree to record their diet at home (P). 2. Agree to self-monitor at home and record results (P). 3. Agree to attend the fifth session at the scheduled time (P). |
| Session 5: reinforcing healthy behaviors | (1) Experience sharing and knowledge review | 1. Report on their experiences (P). 2. Answer the questions on the question cards (C). |
|  | (2) Reinforcing healthy eating | 1. Distinguish between three categories of foods: ① foods that support weight management and can be consumed in larger portions; ② foods that are essential for the body and should be consumed in moderation; ③ foods that interfere with weight management and should be minimized or avoided (P). |
|  | (3) Personalized dietary assessment and guidance | 1. Know if the current diet is appropriate (C). 2. Recognize the importance of dietary management (A). 3. Adhere to dietary management (P). |
|  | (4) Reinforcing regular exercise | 1. 7) Report on physical activity since the last session (P). 2. Recognize the importance of regular exercise (A). 3. Adhere to an exercise plan (P). |
|  | (5) Reinforcing weight management and postpartum follow-up | 1. Report on weight monitoring since the previous session (P). 2. Adhere to weight monitoring and regular follow-up (P). 3. Recognize the importance of adherence to weight management and postnatal follow-up (A). |
|  | (6) Reinforcing breastfeeding | 1. Report on breastfeeding since the previous session (P). 2. Recognize the importance of breastfeeding (A). 3. Adhere to breastfeeding (P). |
|  | (7) Summary | 1. Recognize that regular postnatal follow-up can promote self-management (A). 2. Completed the questionnaire (P). 3. Agree to follow up at the scheduled time and share experiences (P). 4. Know that they are welcome (A). |
| Total number of items | 56 | 189 |

Gestational diabetes mellitus, GDM; type 2 diabetes mellitus, T2DM; body mass index, BMI; cognitive, C; affective, A; psychomotor, P; personalized, P^a^.
