# Supplementary material for: Developing a comprehensive structured program for managing gestational diabetes mellitus and preventing type 2 diabetes mellitus in Chinese women: a multi-method study
Source: Front Endocrinol (Lausanne). 2025 Aug 1;16:1627702. doi: 10.3389/fendo.2025.1627702 (PMC12353735; doi:10.3389/fendo.2025.1627702)
Supplement: Supplementary Figure 1 — PRISMA Flow Diagram. [file DataSheet1.zip › Table 10.docx]

**Supplementary Table 10** The application of motivation-enhancing strategies in the program.

| **Motivation component** | **Motivation strategy** | **The application of motivation-enhancing strategies in the program** |
| --- | --- | --- |
| **Attention** | **Concreteness:** represent the content in a visual manner or provide relevant examples, anecdotes, biographies, etc., to facilitate learners' understanding of key and difficult concepts. | Each course in the program is equipped with corresponding teaching posters that display core educational content, such as the OGTT process, insulin's glucose-lowering effects, characteristics of glucose metabolism during pregnancy, complications of GDM, symptoms of hypoglycemia, blood glucose monitoring methods, BMI classification, insulin injection methods and sites, characteristics of insulin action, symptoms of neonatal hypoglycemia, postpartum blood glucose changes, and postpartum exercises. This method aims to visualize and concretize abstract concepts through vivid and clear images, helping women with GDM better understand complex relationships, and thereby increasing their interest in learning. |
|  | **Variability:** change the presentation of information (such as teaching methods, teaching formats, teaching media, teaching materials, etc.) and interactive styles (such as tone of voice, body movements, demonstration style, etc.) involved in the teaching process. | (1) Each session uses different teaching methods, including lectures, questions and answers., discussions, demonstrations, group games, exercises, and assignments, aimed at attracting the attention of women with GDM, avoiding monotony in the teaching process, and increasing interaction among women with GDM.  (2) The program combines group-based education with individualized guidance, as well as offline and online education, aiming to adapt to the different needs and interests of women with GDM through flexible teaching methods and to enhance their learning motivation and interest.  (3) The program uses different teaching materials based on the teaching objectives and key points. For example: pre-class knowledge review using question cards, food classification and individual dietary assessment and recommendations using food cards, insulin injection demonstration using insulin pens and needles, urine ketone monitoring demonstration using urine ketone strips, and post-class knowledge consolidation using review questions, etc. The variety of teaching materials aims to enhance the participation and attention of women with GDM in the course through concrete and visual methods.  (4) In creating teaching materials, charts, symbols, and fonts of varying colors, sizes, and thicknesses are used to increase the diversity of information presentation, making the materials visually appealing and, in turn, improving the attention and interest of women with GDM. |
|  | **Humor:** enhance the humor in teaching components, such as the introduction, information presentation, explanation, and summary, through jokes, metaphors, and other techniques. | Use metaphors to enhance the humor in professional knowledge, such as the need for sugar in cells, the role of insulin in lowering blood glucose, the effect of pregnancy on insulin action, insufficient insulin secretion in women with GDM, the interference of obesity on insulin function, the restoration of normal glucose metabolism in healthy pregnant women after placenta delivery, and the continued insufficient insulin secretion in women with GDM after childbirth, to avoid making teaching content obscure and difficult to understand. |
|  | **Inquiry:** use creative skills to trigger learners to break through conventional thinking patterns, offering optional topics, projects, and tasks to stimulate their interest in exploring the content. | In the food classification game, guided by the educator, women with GDM gradually categorize food cards into 8 groups: nearly calorie-free, high in fat, high in alcohol, high in sucrose and glucose, high in starch, high in protein, fruits, and milk. The food classification game aims to stimulate the curiosity and desire for exploration of women with GDM, encouraging them to actively participate in problem-solving, thinking, and exploration, thereby enhancing their interest and motivation in dietary management. |
|  | **Participation:** Use methods such as games, role-playing, and imitation to promote learner engagement. | The program incorporates various interactive activities, such as questions and answers, discussions, demonstrations, and group games, to enhance the participation and engagement of women with GDM. Its aim is to deepen their understanding of the educational content and increase their interest and motivation for learning and behavior change. |
| **Relevance** | **Experience:** clearly indicate the connection between the learned content and the learner's existing experiences, and use their prior experiences to process the learned content (such as making analogies based on past experiences or finding points of interest for the learner). | When creating educational materials, the program uses metaphors to compare professional terms and physiological mechanisms to familiar objects and relationships in the lives of women with GDM. This helps them better understand the teaching content, thereby increasing their interest and motivation to learn. |
|  | **Present worth:** clearly indicates the immediate, intuitive value of the teaching content. | (1) Clearly points out that the main treatments for GDM include lifestyle interventions and insulin therapy, helping women with GDM understand that this course is crucial for the treatment and management of GDM. This, in turn, encourages them to take action to treat and manage GDM.  (2) Clearly emphasizes that dietary adjustments and appropriate physical activity are the cornerstone for controlling blood glucose and weight in women with GDM, helping them understand the importance of diet and physical activity, thereby increasing their motivation to manage diet and physical activity.  (3) Clearly points out that a healthy diet, regular exercise, and weight control are effective strategies for women with a history of GDM to prevent and delay the onset and development of long-term T2DM. This helps women understand the importance of continuing lifestyle management postpartum, thereby increasing their motivation to maintain lifestyle management after delivery. |
|  | **Future usefulness:** clearly points out the connection between the learning content and the learner’s future development, and guides the learner to relate the learning to their future plans. | Clearly points out that with active management, ensuring appropriate weight gain during pregnancy, and keeping blood glucose within normal range, the impact of GDM on both the fetus and the mother can be minimized as much as possible. Linking the self-management of GDM women to future maternal and child health helps increase their motivation for lifestyle management. |
|  | **Need matching:** provide appropriate opportunities to meet important learning needs (such as learning achievement, sense of responsibility, social cooperation, sense of belonging, etc.) through methods such as behavior reinforcement, teaching feedback, and building trust. | Develop teaching objectives and content based on the needs analysis of women with GDM, and assess the acceptability of the program through a pilot test. The program meets the preferences and needs of women with GDM and helps to increase the practicality of the content and the participation of women with GDM. |
|  | **Modeling:** set an example for learners through methods such as peer encouragement and acting as a mentor. | In the final session, the educator invites women with GDM to share their self-management experiences in areas such as diet, exercise, weight control, and breastfeeding. The aim is to guide women in self-reflection and experience exchange, encouraging them to learn from positive role models in self-management. |
|  | **Choice:** learners can independently choose learning strategies and methods. | In the final class, considering the needs of women with GDM and the time pressures after childbirth, the program offered them the option to choose between online and offline learning formats, aiming to increase learning flexibility and enhance the willingness of women with GDM to engage in learning. |
| **Confidence** | **Learning requirements:** the learning materials clearly outline engaging objectives and provide self-assessment tools and evaluation standards based on these objectives. | (1) In the first prenatal session, educators set blood glucose and weight management goals for women with GDM; in the first postnatal session, educators set postnatal weight management goals for the women. During the program, educators teach them self-monitoring methods for blood glucose and weight, as well as the measures required to achieve these goals.  (2) Tools to assess the learning progress of women with GDM are provided, such as GDM knowledge questionnaires, review questions after sessions, and pre-class review question cards. These aim to help women with GDM assess their current understanding. Women with GDM also gain a sense of accomplishment after completing each task, which enhances their confidence in continuing to learn and apply the content in their daily lives.  (3) In the introduction to the first session, the educators clearly state the frequency and dosage of the program and explain that everyone should participate actively, with the aim of helping women with GDM understand the learning rhythm during the first session and assisting them in planning their learning. |
|  | **Difficulty:** organize learning materials according to difficulty, providing learners with progressive challenges. | (1) The teaching materials are designed with consideration of the characteristics of the GDM population and the varying levels of ability among women with different educational backgrounds. They are organized in a clear and easy-to-understand sequence, aiming to reduce the difficulties women with GDM might face due to overly difficult teaching content.  (2) The difficulty of the teaching content progresses gradually, starting with simple concepts and then moving on to more complex relationships and mechanisms. This is designed to ensure that women with GDM can gradually master new knowledge without feeling excessive pressure and to avoid frustration caused by encountering complex content too soon. |
|  | **Expectations:** help learners set achievable goals and point out the effort required to reach those goals, guiding them in forming a learning plan. | (1) Educators provide statements to women with GDM about the possibility of success through effort. For example, with active management, ensuring appropriate weight gain during pregnancy and keeping blood glucose within the normal range, the impact of GDM on both the fetus and mother can be minimized, aiming to help women with GDM build confidence in achieving self-management goals and believe in their ability to control the condition.  (2) Educators create specific diet and exercise plans for women with GDM, making it more actionable to achieve blood glucose and weight management goals and control the condition, thereby reducing the confusion or uncertainty women with GDM may experience when facing self-management goals. |
|  | **Attributions:** attribute the learner's success to effort rather than luck or task ease, and encourage learners to make reasonable attributions about their learning success or failure. | Before each session, set up assessments and guidance for indicators such as blood glucose, weight, and blood pressure. During this process, educators guide women with GDM to make correct attributions. For women who have achieved significant results in managing blood glucose and weight, educators should fully acknowledge their efforts; for women who have made efforts but have not achieved the desired results, educators should guide them not to blame themselves for the failure, and encourage them to maintain a positive mindset and continue to persist and strive. |
|  | **Self-confidence:** allow learners to gradually enhance their independence in learning and applying a skill, learn the skill in a low-risk environment, and apply it in real-life situations. It should also help learners realize that pursuing success allows for failure, and feel good about themselves in the process. | Educators emphasize the importance of effort and progress, highlighting that every small step of improvement is worth encouraging, and make every effort to avoid using negative language. This aims to help women with GDM see the results of their efforts and maintain a positive learning attitude. |
| **Satisfaction** | **Natural consequences:** encourage learners to apply the newly learned skills in real-life situations as much as possible, inspire a sense of pride when completing difficult tasks, and provide opportunities for learners who have completed tasks to assist those who have not. | (1) Encourage women with GDM to apply the new skills learned in the course in real-life environments as much as possible, such as blood sugar monitoring, weight monitoring, urine ketone monitoring, dietary adjustments, physical activity, etc. The goal is to help these women see the direct results of their actions (e.g., blood sugar control, and reasonable weight gain) and boost their confidence in continuing to learn and manage their health.  (2) Affirm any actions or characteristics required for success by women with GDM, so they feel that their efforts are recognized and enhance their motivation to continue striving for self-management. |
|  | **Unexpected rewards:** provide unexpected, unconnected rewards for tasks of interest. | When women with GDM achieve self-management goals, provide them with appropriate rewards to enhance their sense of satisfaction and stimulate their ongoing motivation to apply the lifestyle management strategies learned in their daily lives. |
|  | **Positive outcomes:** provide verbal praise and personal attention, and give timely feedback based on the actual situation. | When women with GDM achieve self-management goals, provide immediate verbal praise to enhance their sense of satisfaction and confidence, promoting their continued motivation for lifestyle management. |
|  | **Negative influences:** avoid using methods such as threats and surveillance to force learners to complete tasks. | Avoid using methods such as threats, punishment, and surveillance to force women with GDM to achieve self-management goals, aiming to create a more supportive and respectful learning and self-management environment. |
|  | **Scheduling:** provide continuous reinforcement when learning new tasks, and provide intermittent reinforcement as the learner becomes more proficient and skilled at the task. | In the prenatal sessions, the time interval between the first and second sessions is short, with the second session scheduled within one week after the first. This aims to provide continuous positive feedback and encouragement to women with GDM, helping them build and maintain confidence and motivation in learning and self-management. In the postpartum sessions, courses are scheduled at 6 weeks and 3 months postpartum, aiming to intermittently reinforce the women’s lifestyle, helping them stay alert in self-management and maintain long-term motivation. |

Gestational diabetes mellitus, GDM; oral glucose tolerance test, OGTT; type 2 diabetes mellitus, T2DM.
