# Supplementary material for: Developing a comprehensive structured program for managing gestational diabetes mellitus and preventing type 2 diabetes mellitus in Chinese women: a multi-method study
Source: Front Endocrinol (Lausanne). 2025 Aug 1;16:1627702. doi: 10.3389/fendo.2025.1627702 (PMC12353735; doi:10.3389/fendo.2025.1627702)
Supplement: Supplementary Figure 1 — PRISMA Flow Diagram. [file DataSheet1.zip › Table 11.pdf]

Supplementary Table 11 The application of nudge strategies in the program.

Nudge strategies	Implications of nudge strategies	Application of nudge strategies in this program	Teaching materials
**Messenger**	We are heavily influenced by who communicates information to us.	Educators emphasize to women with GDM that they are professionally trained in order to establish an authoritative image, thereby enhancing the credibility of the information and helping women more easily trust and adopt the instructional content.	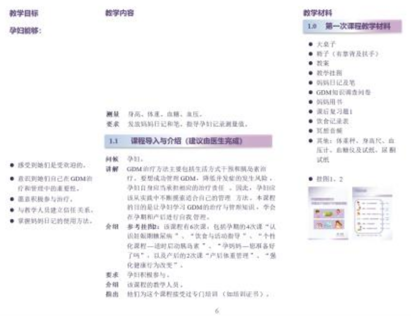 Lesson plan, which shows the step of introducing the educator.
**Simplification**	Complexity in programs significantly reduces their effectiveness and accessibility, often leading to confusion, increased costs, and decreased participation. Simplifying forms and regulations should be a priority to ensure better outcomes in areas like education, health, finance,	(1) Simplifying complex medical knowledge through metaphorical rhetoric makes it easier for women with GDM to understand – Cells need glucose. Brown represents the intestines, the white squares represent glucose, red represents the blood circulation, the blue "factory" represents the pancreas, the blue "key" represents insulin, yellow "doors" represent the cells, and the white "keyhole" represents the insulin receptor. Starch-rich foods are broken down into individual glucose molecules in the intestines. These glucose molecules then enter the bloodstream, causing blood sugar levels to rise. Once the glucose in the	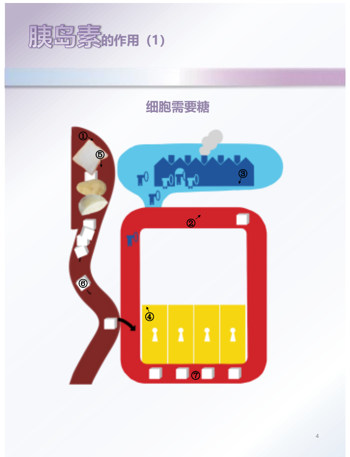

	poverty, and employment.	blood enters the cells, it provides energy for the body. However, glucose cannot enter the cells on its own; therefore, insulin, the "key," is needed to unlock the "door" of the cell.	Teaching poster: cells need glucose.
		(2) Simplifying complex medical knowledge through metaphorical rhetoric makes it easier for women with GDM to understand – Insulin lowers blood glucose. For healthy individuals, the pancreas, as a "factory," has a "chimney" emitting smoke, indicating that it is functioning properly and able to release insulin. Insulin then enters the bloodstream and acts like a "key," fitting into the "keyhole" on the cell and unlocking it, allowing glucose to enter the cell, which results in lower blood glucose levels.	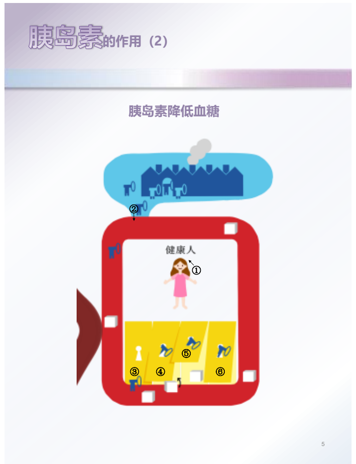 Teaching poster: insulin lowers blood glucose.

		(3) Simplifying complex medical knowledge through metaphorical rhetoric makes it easier for women with GDM to understand – The effect of pregnancy on insulin action. As pregnancy progresses, the placenta secretes substances such as placental lactogen, estrogen, and progesterone. These substances act like "magnets," attracting the "key" and preventing it from functioning properly, which means that more insulin is needed to unlock the cell and allow glucose to enter, thus lowering blood glucose. To meet the increased insulin demand during pregnancy, the "factory" must work harder, as shown in the diagram, with two "chimneys" emitting smoke simultaneously, increasing insulin production. The pancreas "factory" of a healthy pregnant woman can secrete enough insulin through hard work to maintain blood glucose levels within the normal range.	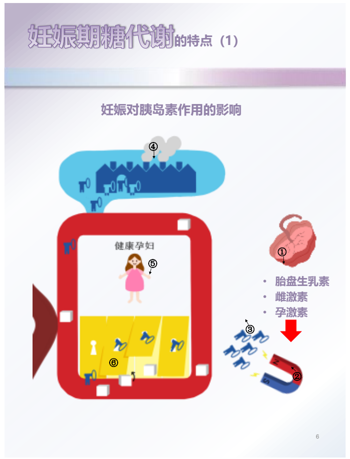 Teaching poster: the effect of pregnancy on insulin action.

		(4) Simplifying complex medical knowledge through metaphorical rhetoric makes it easier for women with GDM to understand – Insulin secretion is inadequate in women with GDM, and obesity interferes with the glucose-lowering effects of insulin. GDM pregnant women have some damage to their "factory," which cannot function properly, meaning the "factory's" ability to produce insulin is reduced, and it cannot secrete enough insulin to keep blood glucose at normal levels. Additionally, in obesity, the "keyhole" of the cell changes due to the presence of excess fat, requiring more "keys" to open the cell and allow glucose to enter, which means the demand for insulin further increases. To meet the high demand for insulin, the "factory" must work even harder, but because part of the "factory" is damaged and cannot function properly, it cannot provide enough insulin to open the cell and allow glucose to enter, resulting in persistently high blood glucose levels.	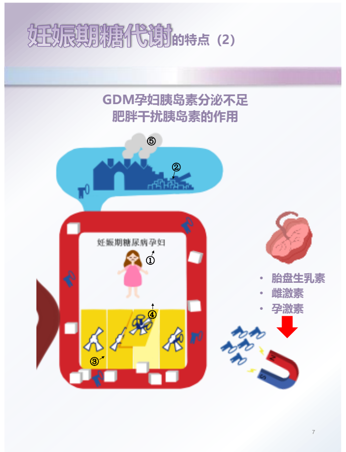<br>Teaching poster: insulin secretion is inadequate in women with GDM, and obesity interferes with the glucose-lowering effects of insulin.

		(5) Simplifying complex medical knowledge through metaphorical rhetoric makes it easier for women with GDM to understand – The glucose metabolism of healthy pregnant women returns to normal after the placenta is delivered. The substances secreted by the placenta, such as placental lactogen, estrogen, and progesterone, disappear with the delivery of the placenta, and the insulin "keys" produced by the pancreas "factory" are no longer attracted. For healthy postpartum women, the "factory" works normally, meaning that one "chimney" emitting smoke is sufficient to meet the body’s insulin needs.	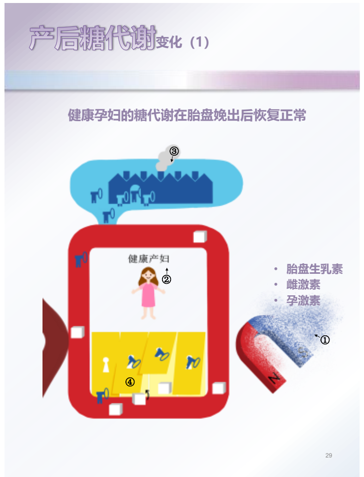<br><br>Teaching poster: the glucose metabolism of healthy pregnant women returns to normal after the placenta is delivered.

<table>
<tr>
<td></td>
<td></td>
<td>(6) Simplifying complex medical knowledge through metaphorical rhetoric makes it easier for women with GDM to understand – Women with GDM may continue to experience insulin deficiency after delivery, and obesity interferes with insulin’s action. Due to partial damage to the pancreas "factory" in women with GDM, the ability to produce insulin is reduced. Even though the "magnet" that attracts the "key" disappears, the insulin produced by the "factory" may still not fully meet the body's needs, resulting in abnormal blood sugar levels postpartum. Additionally, in obesity, the "keyhole" of the cell changes due to excess fat, requiring more "keys" to open the cell and allow glucose to enter, which means the demand for insulin increases. However, because part of the "factory" is damaged and cannot function properly, it cannot provide enough insulin, leading to abnormal blood sugar elevation. Obesity increases the risk of postpartum hyperglycemia.</td>
<td>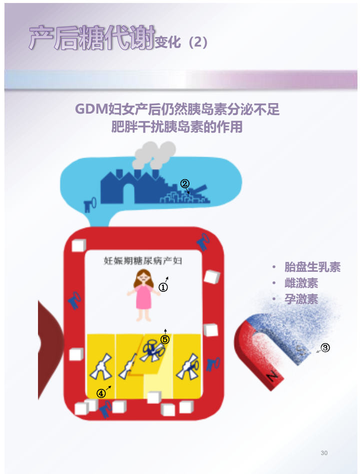

Teaching poster: women with GDM may continue to experience insulin deficiency after delivery, and obesity interferes with insulin’s action.</td>
</tr>
</table>

		(7) Simplifying the wide variety of food types. The program categorizes food into eight groups: almost calorie-free, high in fat, high in alcohol, high in sucrose and glucose, high in starch, high in protein, fruits, and milk, and further simplifies them into three categories: foods that can be eaten more (almost calorie-free), foods to avoid (high in fat, high in alcohol, and high in sucrose and glucose), and foods to eat in moderation (high in starch, high in protein, fruits, and milk). This simplification of food types reduces the cognitive load for women with GDM, helping them more easily understand and remember the health value of each food type, thus enabling them to make healthier dietary choices.	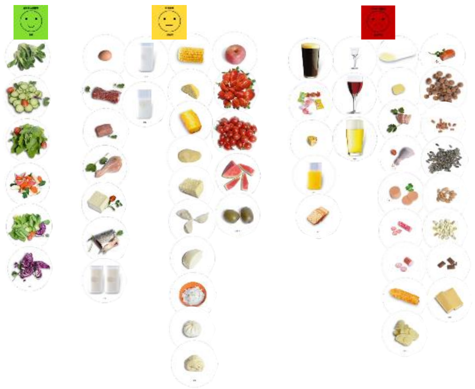<br>Eight food groups and three categories.
		(8) Simplifying food quantification methods. The research team created 55 circular, colorful food cards with a diameter of 18.5 cm. Except for the vegetable card, which has approximately 0 kcal, all other food cards contain 100 kcal per card. When creating the food cards, the size of the food was not magnified or reduced; the size of the food depicted on the cards was the same as the actual food size. Therefore, in real-life situations, by simply looking at the food images on the cards, individuals can intuitively perceive the size of the food represented by one card (100 kcal). Individualized dietary advice for women with GDM is provided based on these food cards. This simplified method of food	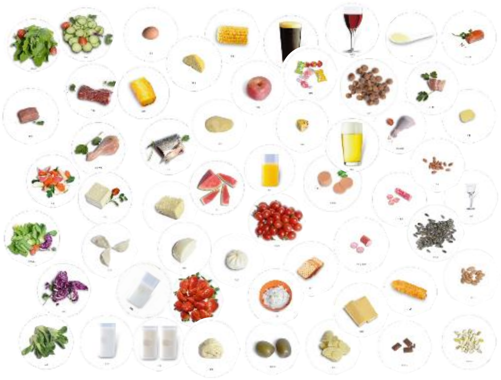<br>Fifty-five food cards.

		quantification helps reduce the cognitive burden on women with GDM regarding the precise weighing of food, making it easier for them to manage their diet.	
		(9) Simplifying complex dietary management principles. Based on food classification, this program further proposes simple and practical golden dietary rules for weight management, including: ① water does not cause weight gain; ② the diet should primarily consist of fresh vegetables, with moderate amounts of staple foods and meats; and ③ be cautious with sugar, fat, and alcohol. By simplifying the complex principles of dietary management, the program provides easy-to-remember and actionable guidance for women with GDM, helping them achieve better dietary control.	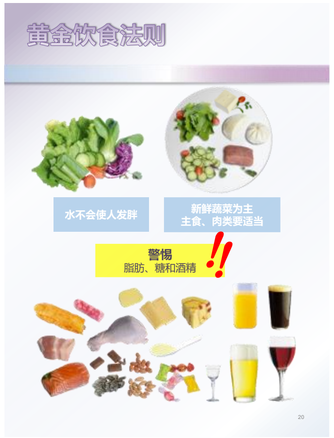<br>Teaching poster: golden dietary rules for weight management.
**Salience**	Our attention is drawn to what is novel and seems relevant to us.	（1）In the food categorization game, green cards represent "foods that can be eaten in larger quantities," red cards represent "foods that should be avoided," and yellow cards represent "foods to be consumed in moderation." This simple and attractive classification helps capture the attention of women with GDM, thus influencing their dietary choices.	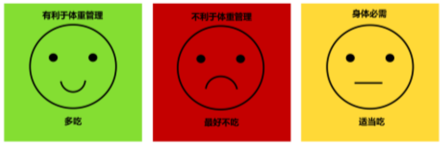<br>Classification cards.

		(2) In the three golden dietary rules poster for weight management, rule 1 "water does not cause weight gain " and rule 2 "the diet should primarily consist of fresh vegetables, with moderate amounts of staple foods and meats" use a light blue background, consistent with the overall style of the teaching materials. However, rule 3 "be cautious with sugar, fat, and alcohol" uses a bright yellow background to attract the attention of women with GDM through its prominence.	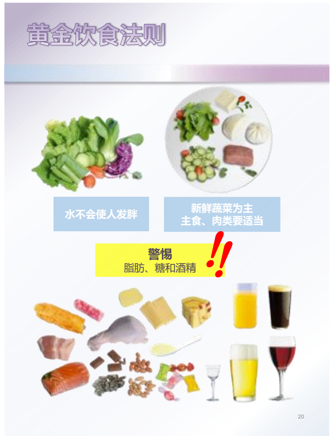<br>Teaching poster: golden dietary rules for weight management.
**Affect**	Our emotional associations can powerfully shape our actions.	In the food classification cards, green cards are paired with a "smiley face," red cards with a "sad face," and yellow cards with a "neutral face." These emoticons help evoke emotional responses in women with GDM, thereby enhancing their understanding of food classification and guiding them toward healthier dietary choices.	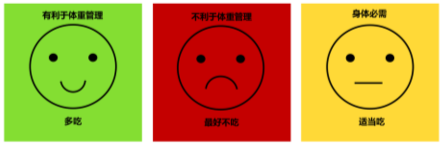<br>Classification cards

**Position**	Changing the position, proximity, or accessibility of products or objects affects our choices.	In the food card classification game, healthy foods (those that can be eaten in larger quantities) are placed closest to the women with GDM, while unhealthy foods (those that should be avoided) are placed furthest away, making healthy foods more noticeable and encouraging women to focus on and choose healthier options.	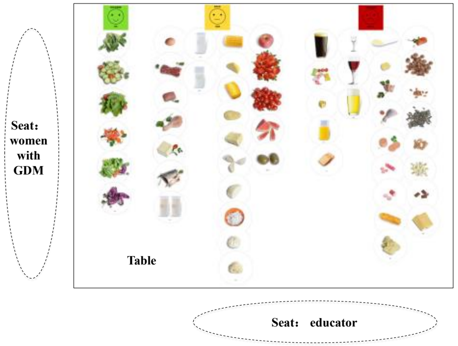<br>Diagram of food card placement and seating.
**Priming**	Our acts are often influenced by sub-conscious cues.	(1) In the poster showing the benefits of breastfeeding, images depicting pre-pregnancy, pregnancy, early postpartum, and postpartum body recovery are used to encourage women with GDM to associate breastfeeding with postpartum weight management, subtly influencing their breastfeeding decisions.	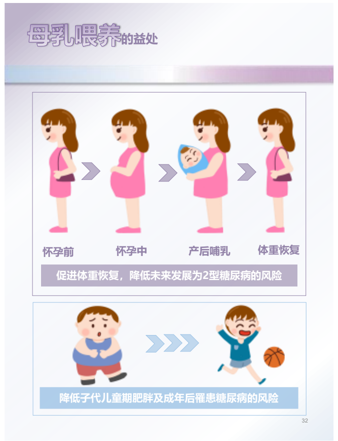<br>Teaching poster: displays images of different stages, including pre-pregnancy, pregnancy, breastfeeding postpartum, and weight

			recovery.
		(2) In the food classification game, the colors of the classification cards are consistent with traffic lights. The goal is to help women with GDM understand and remember food categories by using this familiar signal. It encourages them to associate green cards with foods that can be eaten in larger quantities, red cards with foods that should be avoided, and yellow cards with foods that should be eaten in moderation, thereby influencing their dietary choices.	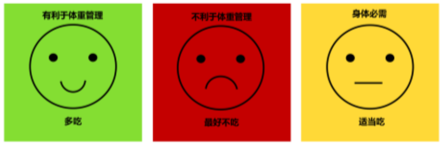 Classification cards
**Commitment**	We seek to be consistent with our public promises and reciprocate acts.	Educators help women with GDM set weight management goals for both pregnancy and postpartum during their first sessions, and ask them to record these goals in their "Mother’s Diary." This approach helps women with GDM clarify their weight management goals, strengthen their sense of commitment and responsibility, and motivate them to take concrete actions to achieve these goals.	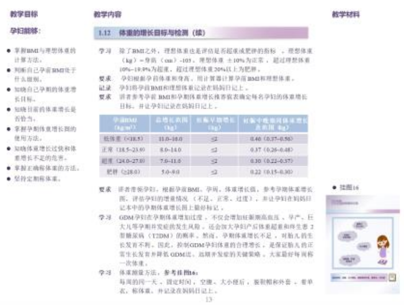<br>Lesson plan: setting goals for weight management during pregnancy.

			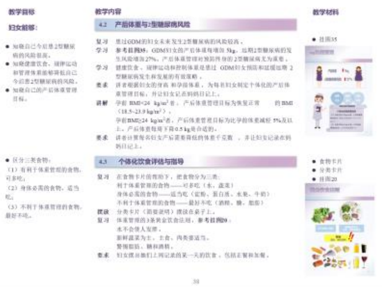<br>Lesson plan: setting goals for weight management in the postpartum period.
**Warnings**	Large fonts, bold letters, and bright colors can be effective in triggering our attention.	(1) In the poster depicting adverse outcomes of GDM on mother and baby, the use of a red lightning icon attracts the attention of women with GDM, highlighting the potential risks of negative pregnancy outcomes and reminding them of the possible adverse consequences of GDM, thus encouraging them to make more cautious and proactive behavioral choices.	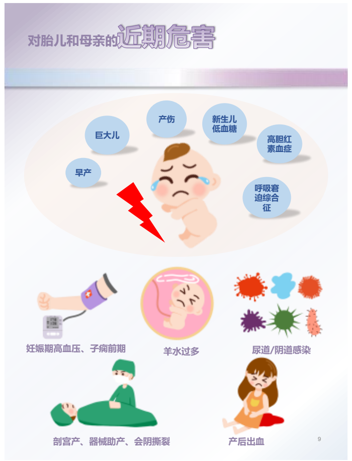<br><br>Teaching poster: adverse effects of GDM on mothers and babies.

		(2) In the poster of the three golden dietary rules for weight management, the use of a red exclamation mark icon draws the attention of women with GDM to Rule 3, "be cautious with sugar, fat, and alcohol," enhancing their risk perception and prompting them to be more cautious in their choices of sugar, fat, and alcohol.	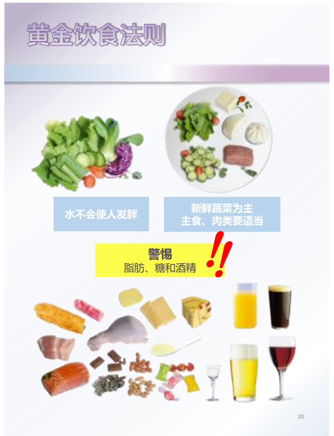<br><br>Teaching poster: three golden dietary rules for weight management.
		(3) In the poster of sugar-free foods, the use of a red lightning icon draws the attention of women with GDM to the potential risks of sugar alcohol, encouraging them to be more cautious in their choices regarding sugar alcohol.	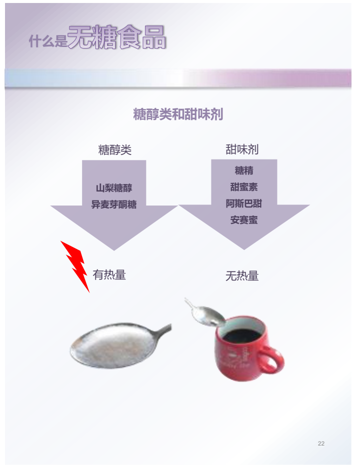

<table>
<tr><td></td><td></td><td></td><td>Teaching poster: sugar-free food.</td></tr>
<tr><td></td><td></td><td>(4) In the poster of physical activity during pregnancy, the use of a red lightning icon draws the attention of women with GDM to the indications for stopping exercise during pregnancy, reminding them to be aware of the potential risks of exercise during pregnancy.</td><td>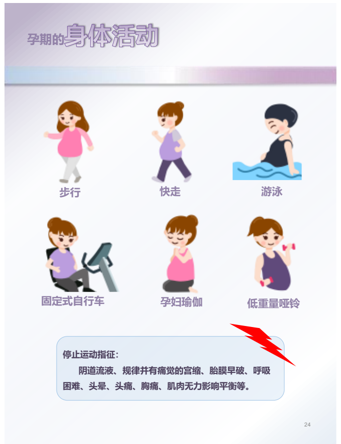

Teaching poster: physical activity during pregnancy.</td></tr>
<tr><td></td><td></td><td>(5) In the poster of postpartum weight management, the use of a red, bold upward arrow highlights the positive correlation between postpartum weight in women with GDM and the risk of T2DM, reminding them to pay attention to this health risk.</td><td>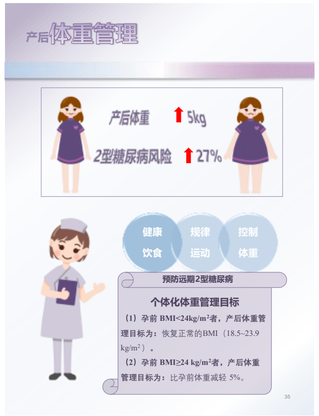
</td></tr>
</table>

			Teaching poster: postpartum weight management.
**Reminders**	Timely reminders help us to overcome inertia, procrastination, and forgetfulness and enable us to engage in certain behaviors.	Post-class review questions, question cards, GDM knowledge questionnaire, mother’s diary, and mother’s handbook serve as reminders by making the teaching content reappear repeatedly, drawing women's attention and encouraging them to consolidate knowledge and adopt expected behaviors.	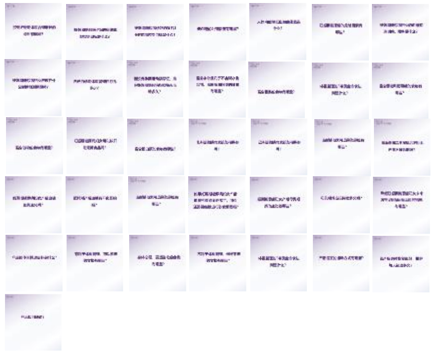 Question cards for pre-class knowledge review. 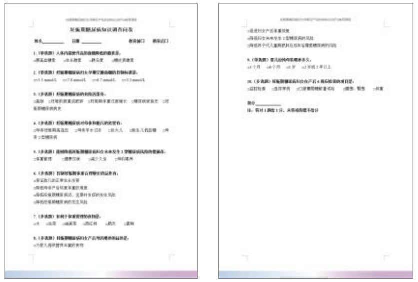 GDM knowledge questionnaire.

			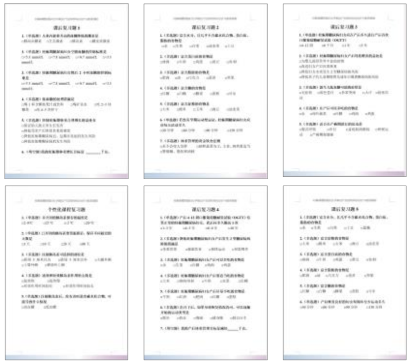<br>Post-class review questions.<br>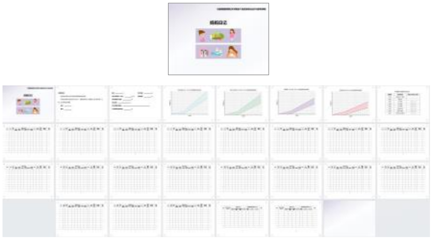<br>Mother’s diary.

			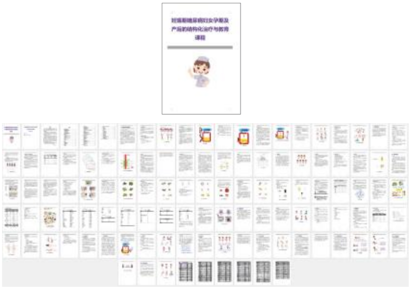<br>Mother’s handbook.

Gestational diabetes mellitus, GDM.
