# Supplementary material for: Developing a comprehensive structured program for managing gestational diabetes mellitus and preventing type 2 diabetes mellitus in Chinese women: a multi-method study
Source: Front Endocrinol (Lausanne). 2025 Aug 1;16:1627702. doi: 10.3389/fendo.2025.1627702 (PMC12353735; doi:10.3389/fendo.2025.1627702)
Supplement: Supplementary Figure 1 — PRISMA Flow Diagram. [file DataSheet1.zip › Table 12.docx]

**Supplementary Table 12** The basic information of experts in the expert meeting.

| **Number** | **Gender** | **Age** | **Education level** | **Years of working** | **Professional title** | **Research field** |
| --- | --- | --- | --- | --- | --- | --- |
| 1 | Female | 55 | Doctoral degree | 33 | Professor | Chronic Disease Management |
| 2 | Female | 38 | Doctoral degree | 8 | Associate Researcher | Psychology |
| 3 | Female | 51 | Doctoral degree | 26 | Professor | Public health and health education |
| 4 | Male | 45 | Doctoral degree | 14 | Associate Professor | Chronic diseases and nutrition |
| 5 | Female | 49 | Doctoral degree | 20 | Professor | Obstetrics |
| 6 | Female | 44 | Master's degree | 17 | Associate Chief Senior Nurse | Obstetrics |
