# Supplementary material for: Developing a comprehensive structured program for managing gestational diabetes mellitus and preventing type 2 diabetes mellitus in Chinese women: a multi-method study
Source: Front Endocrinol (Lausanne). 2025 Aug 1;16:1627702. doi: 10.3389/fendo.2025.1627702 (PMC12353735; doi:10.3389/fendo.2025.1627702)
Supplement: Supplementary Figure 1 — PRISMA Flow Diagram. [file DataSheet1.zip › Table 13.docx]

**Supplementary Table 13** Experts' suggestions on the program.

| **Teaching materials** | **Experts’ suggestions** |
| --- | --- |
| Lesson plan | (1) To enhance the readability of the title, it is recommended that the abbreviation "STEP" on the lesson plan cover be replaced with "Structured Treatment and Education Program."  (2) It is recommended to standardize the terminology used to refer to women with GDM in the lesson plans. For example, in the pregnancy lesson plan, it is suggested to use the term "pregnant women," while in the postpartum lesson plan, the term "women" should be used.  (3) In the section on complications of GDM in the first session, congenital malformations and stillbirths are more common among women with pregestational diabetes. However, since this program specifically focuses on women with GDM, it is recommended to exclude these two adverse outcomes.  (4) In the fourth session, only the range of weight management goals for women with GDM was provided, without specifying how many kilograms they need to lose to achieve these goals. Therefore, it is recommended to include a step where educators calculate how many kilograms each woman with GDM needs to lose postpartum to reach the weight management goal. |
| Teaching posters | (1) The abbreviation "STEP" may be difficult for women with GDM to understand. It is recommended that the abbreviation be replaced with "Structured Treatment and Education Program" on the cover of the teaching posters.  (2) Since congenital malformations and stillbirths are more common in women with pregestational diabetes, but this program focuses specifically on women with GDM, it is recommended to remove these two outcomes from page 9 of the teaching poster.  (3) The illustration of insulin injection sites on page 25 of the teaching poster depicts a male figure. Given that this program is specifically designed for women with GDM, it is recommended to replace this image with a female figure.  (4) The poster on page 32 presents the benefits of breastfeeding. It is recommended to add an image of a mother holding her baby on this page to help women with GDM more intuitively experience the intimacy and naturalness of breastfeeding. |
| Mother’s handbook | (1) The abbreviation "STEP" may be difficult for women with GDM to understand. It is recommended that the abbreviation be replaced with "Structured Treatment and Education Program" on the cover of the mother’s handbook.  (2) Since congenital malformations and stillbirths are more common in women with pregestational diabetes, but this program focuses specifically on women with GDM, it is recommended to remove these two outcomes from page 12 of the mother’s handbook.  (3) According to the World Health Organization and China's guidelines for the prevention and treatment of type 2 diabetes mellitus, it is recommended that the phrase "male waist circumference ≥85 cm and female waist circumference ≥80 cm are the cut-off points for central obesity" on page 16 of the mother's handbook be changed to "male waist circumference ≥90 cm and female waist circumference ≥85 cm are the cut-off points for central obesity." |
| Question cards | (1) It is recommended to revise the question "When should ketone bodies be tested in women with GDM?" to "When should urine ketone bodies be tested in women with GDM?"  (2) It is recommended to revise the question 'What are the criteria for fasting glucose control in women with GDM?' to 'What are the goals for fasting glucose control in women with GDM?  (3) It is recommended to revise the question "What is the hormone in the body that lowers elevated blood glucose?" to "What is the hormone in the body that lowers blood glucose?" |
| Post-class review questions | It is recommended to revise the question "My postpartum weight management goal is " to "My postpartum weight management goal is to lose kilograms" in question 7 of post-class review question 4. |
| Mother's file | (1) It is recommended to add units for weight, blood glucose, blood pressure, and total calorie intake.  (2) In the OGTT result recording section, it is recommended to add fields to record blood glucose values at different time points. |
| Mother’s diary | It is recommended to add units for height, pre-pregnancy weight, and ideal weight." |

Gestational diabetes mellitus, GDM; oral glucose tolerance test, OGTT; structured treatment and education program, STEP.
