# Supplementary material for: Developing a comprehensive structured program for managing gestational diabetes mellitus and preventing type 2 diabetes mellitus in Chinese women: a multi-method study
Source: Front Endocrinol (Lausanne). 2025 Aug 1;16:1627702. doi: 10.3389/fendo.2025.1627702 (PMC12353735; doi:10.3389/fendo.2025.1627702)
Supplement: Supplementary Figure 1 — PRISMA Flow Diagram. [file DataSheet1.zip › Table 14.docx]

**Supplementary Table 14** Trial session process and feedback.

| **Sessions** | **Pilot session 1** | | **Pilot session 2** | | **Pilot session 3** | |
| --- | --- | --- | --- | --- | --- | --- |
|  | **Duration** | **Feedback** | **Duration** | **Feedback** | **Duration** | **Feedback** |
| Session 1 | 72minutes | (1) A rapid rate of speech may result in unclear information delivery, potentially hindering the comprehension of women with GDM. Conversely, a slow rate of speech may lead to a loss of participant engagement. Therefore, it is recommended that educators maintain an optimal speaking pace to ensure that women with GDM can hear and effectively comprehend the instructional content. (2) It is recommended that educators use gestures or a pointer to highlight relevant key points on teaching posters during instructional sessions, in order to help women with GDM maintain pace with the teaching progression. (3) When presenting urinary ketone test strips to women with GDM, it is recommended that educators clarify that these strips are for educational purposes only, or label the package as 'for educational purposes.' | 76 minutes | No new suggestions. |  |  |
| Session 2 | 65 minutes | (1) When using food cards for food categorization activities, it is recommended that educators explain the key nutritional characteristics of each food while placing the cards. This helps women with GDM better understand the nutrient content of each food and its role in glycemic control, thereby enhancing their understanding and mastery of healthy dietary patterns. (2) During individualized dietary assessments, since women with GDM may encounter difficulties when using food cards to display their daily intake, it is recommended that educators provide guidance and support to assist them in selecting and arranging the food cards appropriately, enabling a comprehensive evaluation of their dietary structure. | 80 minutes | Women with GDM are predominantly young adults, and although their educational backgrounds may vary, they generally possess strong cognitive abilities. Therefore, it is recommended that educators use language that is both approachable and professional when communicating with them while avoiding overly simplified expressions. | 83 minutes | No new suggestions. |
| Session 2P | 42 minutes | When presenting insulin pens and needles to women with GDM, it is recommended that educators clarify that these instruments are for educational purposes only, or label the package as 'for educational purposes.' | 45 minutes | No new suggestions. |  |  |
| Session 3 | 48 minutes | No problems were found with the scientificity and rationality of the session. |  |  |  |  |
| Session 4 | 43 minutes | No problems were found with the scientificity and rationality of the session. |  |  |  |  |
| Session 5 | 45 minutes | No problems were found with the scientificity and rationality of the session. |  |  |  |  |

Gestational diabetes mellitus, GDM; personalized, P.
