# Supplementary material for: Developing a comprehensive structured program for managing gestational diabetes mellitus and preventing type 2 diabetes mellitus in Chinese women: a multi-method study
Source: Front Endocrinol (Lausanne). 2025 Aug 1;16:1627702. doi: 10.3389/fendo.2025.1627702 (PMC12353735; doi:10.3389/fendo.2025.1627702)
Supplement: Supplementary Figure 1 — PRISMA Flow Diagram. [file DataSheet1.zip › Table 15.docx]

**Supplementary Table 15** The basic information, pregnancy weight gain, and postpartum blood glucose of participants in the pilot test.

| **Number** | **Age (years)** | **Marital status** | **Ethnic group** | **Educational level** | **Family history of diabetes mellitus** | **Pregnancy history** | **Gestational weeks/** **weeks after delivery** |
| --- | --- | --- | --- | --- | --- | --- | --- |
| 1 | 28 | Married | Manchu ethnicity | Bachelor degree | Yes | G0, P0 | 26 weeks of gestation |
| 2 | 39 | Married | Han ethnicity | Bachelor degree | Yes | G0, P0 | 25 weeks of gestation |
| 3 | 35 | Married | Han ethnicity | Associate degree | No | G0, P0 | 32 weeks of gestation |
| 4 | 43 | Married | Han ethnicity | Master degree | Yes | G0, P0 | 33 weeks of gestation |
| 5 | 31 | Married | Han ethnicity | Master degree | No | G0, P0 | 33 weeks of gestation |
| 6 | 31 | Married | Han ethnicity | Bachelor degree | Yes | G2, P0 | 34 weeks of gestation |
| 7 | 30 | Married | Han ethnicity | Master degree | No | G1, P1 | 6 weeks after delivery |
| 8 | 36 | Married | Han ethnicity | Master degree | Yes | G2, P2 | 7 weeks after delivery |
| **Number** | **Height (cm)** | **Weight before pregnancy (kg)** | **BMI before pregnancy (kg/m^2^)** | **Pregnancy weight gain (kg)** | **OGTT at 4-12 weeks postpartum** | **OGTT 0-minute blood glucose (mmol/L )** | **OGTT 120-minute blood glucose (mmol/L )** |
| 1 | 159.0 | 63.0 | 24.9 | 10.5 | Yes | 5.1 | 7.0 |
| 2 | 152.0 | 49.0 | 21.2 | 13.0 | Yes | 6.2 | 9.4 |
| 3 | 160.0 | 71.0 | 27.7 | 8.5 | No | - | - |
| 4 | 152.0 | 58.0 | 25.1 | 10.0 | Yes | 4.6 | 9.6 |
| 5 | 170.0 | 63.0 | 21.8 | 15.0 | Yes | 4.6 | 4.4 |
| 6 | 160.0 | 65.5 | 25.6 | 12.5 | Yes | 4.5 | 8.2 |
| 7 | 160.0 | 50.0 | 19.5 | 9.0 | Yes | 4.7 | 8.7 |
| 8 | 167.0 | 64.0 | 22.9 | 9.5 | yes | 5.5 | 8.4 |

Gravidity, G; parity, P; body mass index, BMI; oral glucose tolerance test, OGTT.
