# Supplementary material for: Developing a comprehensive structured program for managing gestational diabetes mellitus and preventing type 2 diabetes mellitus in Chinese women: a multi-method study
Source: Front Endocrinol (Lausanne). 2025 Aug 1;16:1627702. doi: 10.3389/fendo.2025.1627702 (PMC12353735; doi:10.3389/fendo.2025.1627702)
Supplement: Supplementary Figure 1 — PRISMA Flow Diagram. [file DataSheet1.zip › Table 16.docx]

**Supplementary Table 16** Feedback from participants in the pilot test.

| **Number** | **Examples of feedback from women with GDM** |
| --- | --- |
| 1 | "I think the content, format, and duration of the course are fine and everything is quite appropriate. I learned a lot of things I didn't know before. It's beneficial for us to learn more about these things.” |
| 2 | "I think the course is great, and I've learned a lot of useful information from it. Shortly after being diagnosed with GDM, I visited the nutrition department. The doctor spoke very quickly, and I didn't fully understand what I was supposed to do. This course goes into a lot of detail and explains the causes and consequences of GDM, which I find very helpful." |
| 3 | "When I first started using insulin, my doctor didn't explain much to me because she was busy. This course helped clarify some of my doubts and gave me a better understanding of insulin and GDM. I was able to understand all the content, and the duration of the course was appropriate." |
| 4 | "The course was very helpful in increasing my knowledge, and the format was good as well." |
| 5 | "Some of the information has really changed my understanding. For example, I used to think that all the nutrients were in the soup, so I could just drink the soup without eating the meat. After taking the course, I learned that the soup is mostly water, fat, and salt and that the nutrients are actually in the meat. I now know that I should eat the meat while drinking the soup and choose soups with less oil and salt. Overall, I'm satisfied with the course." |
| 6 | "The course was very important in increasing my knowledge. Even though I had been paying attention to my diet before, I couldn't tell the difference between a lot of things. After the course, I now have a clearer understanding of how to manage my diet." |
| 7 | "I think the course is really good. I didn't know much about diabetes before, but I’ve always known that a healthy diet is important for the body. While it's easy to find information online, it can be too fragmented. Taking this course helped me learn a lot of information in a more systematic way." |
| 8 | The course gave me a new understanding of the relationship between GDM and T2DM, and I now know what to do to prevent T2DM and follow-up. Overall, the course was great, and the duration and teaching methods were appropriate." |

Gestational diabetes mellitus, GDM; type 2 diabetes mellitus, T2DM.
