# Supplementary material for: Developing a comprehensive structured program for managing gestational diabetes mellitus and preventing type 2 diabetes mellitus in Chinese women: a multi-method study
Source: Front Endocrinol (Lausanne). 2025 Aug 1;16:1627702. doi: 10.3389/fendo.2025.1627702 (PMC12353735; doi:10.3389/fendo.2025.1627702)
Supplement: Supplementary Figure 1 — PRISMA Flow Diagram. [file DataSheet1.zip › Table 1.DOCX]

**Supplementary table 1** Search strategies for needs analysis.

| **Databese** | **Search strategies** |
| --- | --- |
| Pubmed | ("Health Education"[MeSH Terms] OR "Patient Education as Topic"[MeSH Terms] OR "Health Education"[Title/Abstract] OR "Patient Education"[Title/Abstract]) AND ("diabetes, gestational"[MeSH Terms] OR "gestational diabet*"[Title/Abstract] OR "GDM"[Title/Abstract] OR (("Diabetes Mellitus"[MeSH Terms] OR "diabet*"[Title/Abstract]) AND ("Pregnant Women"[MeSH Terms] OR "pregnan*"[Title/Abstract]))) AND ((("Needs Assessment"[MeSH Terms] OR "Health Services Needs and Demand"[MeSH Terms] OR "Patient Preference"[MeSH Terms] OR "patient perspective*"[Title/Abstract] OR "patient priorit*"[Title/Abstract]) AND "expectation*"[Title/Abstract]) OR "prefer*"[Title/Abstract] OR "needs"[Title/Abstract] OR "demand"[Title/Abstract]) |
| Embase | ('pregnancy diabetes mellitus'/exp OR 'pregnancy diabetes mellitus' OR 'gestational diabet*':ab,ti OR gdm:ab,ti OR (('diabetes mellitus'/exp OR 'diabetes mellitus' OR diabet*:ab,ti) AND ('pregnant woman'/exp OR 'pregnant woman' OR pregnan*:ab,ti))) AND ('health education'/exp OR 'patient education'/exp OR 'health education':ab,ti OR 'patient education':ab,ti) AND ('needs assessment'/exp OR 'patient preference'/exp OR 'health services needs and demand':ab,ti OR 'patient perspective*':ab,ti OR 'patient priorit*':ab,ti OR expectation*:ab,ti OR prefer*:ab,ti OR needs:ab,ti OR demand:ab,ti) |
| PsycINFO | S1 SU “Needs Assessment” OR Su “Health Services Needs and Demand” OR SU “Patient Preference”OR AB “patient perspective*” OR AB “patient priorit*” OR AB expectation* OR AB prefer* OR AB needs OR AB demand  S2 SU “Health Education” OR SU “PatientEducation” OR AB “Health Education” OR AB “Patient Education”  S3 SU “Diabetes Mellitus, Gestational” OR AB “Gestational Diabet*” OR AB GDM OR ((SU “Diabetes Mellitus” OR AB Diabet*) AND (SU “Expectant Mothers” OR AB Pregnan*))  S4 S1 AND S2 AND S3 |
| CINAHL | S1 SU “Needs Assessment” OR Su “Health Services Needs and Demand” OR SU “Patient Preference”OR AB “patient perspective*” OR AB “patient priorit*” OR AB expectation* OR AB prefer* OR AB needs OR AB demand  S2 SU “Health Education” OR SU “PatientEducation” OR AB “Health Education” OR AB “Patient Education”  S3 SU “Diabetes Mellitus, Gestational” OR AB “Gestational Diabet*” OR AB GDM OR ((SU “Diabetes Mellitus” OR AB Diabet*) AND (SU “Expectant Mothers” OR AB Pregnan*))  S4 S1 AND S2 AND S3 |
| Cochrane central register of controlled trials | #1 MeSH descriptor: [Diabetes, Gestational] explode all tree  #2 (Gestational Diabet*):ti  #3 MeSH descriptor: [Diabetes Mellitus] explode all trees  #4 (Diabet*):ti,ab,kw  #5 (Pregnan*):ti,ab,kw  #6 MeSH descriptor: [Pregnant Women] explode all trees  #7 #3 OR #4  #8 #5 OR #6  #9 #1 OR #2 OR (#7 AND #8)  #10 MeSH descriptor: [Patient Education as Topic] explode all trees  #11 MeSH descriptor: [Health Education] explode all trees  #12 (Health Education):ti,ab,kw OR (Patient Education):ti,ab,kw  #13 #10 OR #11 OR #12  #14 MeSH descriptor: [Needs Assessment] explode all trees  #15 MeSH descriptor: [Health Services Needs and Demand] explode all trees  #16 MeSH descriptor: [Patient Preference] explode all trees  #17 (patient perspective*):ti,ab,kw OR (patient priorit*):ti,ab,kw OR (expectation*):ti,ab,kw OR (prefer*):ti,ab,kw OR (needs):ti,ab,kw OR (demand):ti,ab,kw  #18 #14 OR #15 OR #16 OR #17  #19 #18 AND #13 AND #9 |
