# Supplementary material for: Developing a comprehensive structured program for managing gestational diabetes mellitus and preventing type 2 diabetes mellitus in Chinese women: a multi-method study
Source: Front Endocrinol (Lausanne). 2025 Aug 1;16:1627702. doi: 10.3389/fendo.2025.1627702 (PMC12353735; doi:10.3389/fendo.2025.1627702)
Supplement: Supplementary Figure 1 — PRISMA Flow Diagram. [file DataSheet1.zip › Table 2.DOCX]

**Supplementary Table 2** Quality assessment of the qualitative studies and qualitative component in mixed-methods studies.

| **Number** | **Author/year** |  |  |  |  |  |  |  |  |  |  | **Overall** |
| --- | --- | --- | --- | --- | --- | --- | --- | --- | --- | --- | --- | --- |
| 1 | Draffin et al. 2016 | Unclear | Yes | Yes | Yes | Yes | No | No | Yes | Yes | Yes | Included |
| 2 | Oza-Frank et al. 2018 | Unclear | Yes | Yes | Yes | Yes | No | No | Yes | Yes | Yes | Included |
| 3 | Faal et al. 2022 | Unclear | Yes | Yes | Yes | Yes | No | No | Yes | Yes | Yes | Included |
| 4 | Kolivand et al. 2018 | Unclear | Yes | Yes | Yes | Yes | No | No | Yes | Yes | Yes | Included |
| 5 | Khooshehchin et al. 2016 | Unclear | Yes | Yes | Yes | Yes | No | No | Yes | Yes | Yes | Included |
| 6 | Hewage, S. 2020 | Unclear | Yes | Yes | Yes | Yes | No | No | Yes | Yes | Yes | Included |

1. Is there congruity between the stated philosophical perspective and the research methodology?
2. Is there congruity between the research methodology and the research question or objectives?
3. Is there congruity between the research methodology and the methods used to collect data?
4. Is there congruity between the research methodology and the representation and analysis of data?
5. Is there congruity between the research methodology and the interpretation of results?
6. Is there a statement locating the researcher culturally or theoretically?
7. Is the influence of the researcher on the research, and vice-versa, addressed?
8. Are participants, and their voices, adequately represented?
9. Is the research ethical according to current criteria or, for recent studies, and is there evidence of ethical approval by an appropriate body?
10. Do the conclusions drawn in the research report flow from the analysis, or interpretation, of the data?
