# Supplementary material for: Developing a comprehensive structured program for managing gestational diabetes mellitus and preventing type 2 diabetes mellitus in Chinese women: a multi-method study
Source: Front Endocrinol (Lausanne). 2025 Aug 1;16:1627702. doi: 10.3389/fendo.2025.1627702 (PMC12353735; doi:10.3389/fendo.2025.1627702)
Supplement: Supplementary Figure 1 — PRISMA Flow Diagram. [file DataSheet1.zip › Table 3.DOCX]

**Supplementary Table 3** Quality assessment of the quantitative studies and quantitative component in mixed-methods studies.

| **Number** | **Author/year** |  |  |  |  |  |  |  |  |  | **Overall** |
| --- | --- | --- | --- | --- | --- | --- | --- | --- | --- | --- | --- |
| 1 | Sayakhot et al. 2016 | No | Yes | Yes | Yes | Yes | Yes | Yes | Yes | Yes | Included |
| 2 | Alexandra Friedman et al. 2016 | No | Unclear | Unclear | Yes | Unclear | Yes | Yes | Yes | Unclear | Included |
| 3 | Lapolla et al. 2012 | Yes | Unclear | Unclear | Yes | Yes | Yes | Yes | Yes | Unclear | Included |
| 4 | Hewage et al. 2020 | Yes | Unclear | Unclear | Yes | Yes | Yes | Yes | Yes | Unclear | Included |
| 5 | Cheng et al. 2022 | No | Unclear | Unclear | Yes | Yes | Yes | Yes | Yes | Unclear | Included |

1. Was the sample frame appropriate to address the target population?
2. Were study participants sampled in an appropriate way?
3. Was the sample size adequate?
4. Were the study subjects and the setting described in detail?
5. Was the data analysis conducted with sufficient coverage of the identified sample?
6. Were valid methods used for the identification of the condition?
7. Was the condition measured in a standard, reliable way for all participants?
8. Was there an appropriate statistical analysis?
9. Was the response rate adequate, and if not, was the low response rate managed appropriately?
