# Supplementary material for: Developing a comprehensive structured program for managing gestational diabetes mellitus and preventing type 2 diabetes mellitus in Chinese women: a multi-method study
Source: Front Endocrinol (Lausanne). 2025 Aug 1;16:1627702. doi: 10.3389/fendo.2025.1627702 (PMC12353735; doi:10.3389/fendo.2025.1627702)
Supplement: Supplementary Figure 1 — PRISMA Flow Diagram. [file DataSheet1.zip › Table 5.DOCX]

**Supplementary Table 5** Needs and preferences of women with GDM in the systematic review.

| [**Theme**](javascript:;) | **Category** | **Subcategory** |
| --- | --- | --- |
| GDM-related knowledge | Basic knowledge | |
|  | Diagnostic criteria | |
|  | Risk factors | |
|  | The short- and long-term impacts on mothers and infants | |
|  | Key factors in the occurrence and management of GDM | |
|  | The relationship between GDM and T2DM | |
|  | The importance of preventing T2DM | |
| Treatment of GDM | Diet management | Methods of food selection, portioning methods |
|  |  | Meal distribution methods |
|  |  | Healthy cooking methods |
|  |  | Individualized dietary guidance |
|  | Physical activity | The importance of exercise during pregnancy |
|  |  | Guidelines for physical activity during pregnancy |
|  | Antidiabetic medications | The safety of insulin use during pregnancy |
|  |  | Insulin injection methods |
| Glucose monitoring | Blood glucose monitoring and recording, treatment of hypoglycemia | |
|  | Use of blood glucose meters | |
|  | Management of hypoglycemia | |
| Social support | Family support, e.g. husband, parents, parents-in-law, sisters | |
|  | Peer support | |
|  | Support from friend | |
|  | Support from healthcare providers | |
|  | Establishing social interactions and groups | |
| Mental health supports | Professional counseling | |
|  | Education on stress reduction techniques | |
| Postpartum health guidance | Postpartum glucose monitoring and follow-up | Importance of postpartum glucose review |
|  |  | Postpartum glucose monitoring |
|  |  | Postpartum follow up |
|  | Guidance on T2DM prevention | Postpartum dietary guidelines |
|  |  | Postpartum exercise guidelines |
|  | Breastfeeding | The relationship between breastfeeding and T2DM |
|  |  | Guidance on breastfeeding |
|  | Guidance on routine postpartum health care | Newborn care guidance |
|  |  | Postpartum medication guidance |
|  |  | Guidance for preparing for a subsequent pregnancy |
|  | Postpartum psychological counseling | |
| Health Education Settings | The concepts and strategies of health education | Problem-solving orientation |
|  |  | Strategies to improve adherence to lifestyle management |
|  | Health education providers | Physician, diabetes education nurse, internet^*^, dietitian^*^, trained counselor#, diabetes support group^#^ (in descending order of the number of references, with both ^*^ and ^#^ indicating the same number of references) |
|  | Forms of prenatal health education | Face-to-face |
|  |  | Family participation |
|  |  | Detailed health education materials such as books, booklets, pamphlets, brochures, or educational packages |
|  |  | Forms of activities desired with peers: group classes, in person*, talking on the phone*, and text messaging (sort by preference from largest to smallest, * indicates the same preference) |
|  | Forms of postpartum health education | Online maternity school courses, pamphlets, WeChat, telephone consultations, lectures by medical staff, outpatient consultations, and internet (listed in descending order of patient-reported preference in references) |
| Healthcare system | Economic support system for health care such as health benefits for the uninsured | |
|  | Improvement of communication skills and quality of interaction among healthcare providers | Improved communication skills of healthcare providers |
|  |  | Positive interactions with healthcare providers |
|  | The quality of medical services and resource support | High-quality, continuous counseling and education |
|  |  | Well-equipped and advanced medical services |
|  |  | Extended duration of postpartum care, e.g. up to 6 months or even 1 year after delivery |

Gestational diabetes mellitus, GDM; type 2 diabetes mellitus, T2DM.
