# Supplementary material for: Developing a comprehensive structured program for managing gestational diabetes mellitus and preventing type 2 diabetes mellitus in Chinese women: a multi-method study
Source: Front Endocrinol (Lausanne). 2025 Aug 1;16:1627702. doi: 10.3389/fendo.2025.1627702 (PMC12353735; doi:10.3389/fendo.2025.1627702)
Supplement: Supplementary Figure 1 — PRISMA Flow Diagram. [file DataSheet1.zip › Table 6.DOCX]

**Supplementary Table 6** Basic information of women with gestational diabetes mellitus who participated in the needs analysis interviews.

| **Number** | **Age (years)** | **Ethnic group** | **Educational level** | **Marital status** | **Family history of diabetes** | **History of pregnancy** | **Pre-pregnancy BMI (kg/m^2^)** | **Gestational week** |
| --- | --- | --- | --- | --- | --- | --- | --- | --- |
| 1 | 37 | Han ethnicity | Bachelor's degree | Married | Yes | G1 P0 | 25.78 | 29 |
| 2 | 34 | Han ethnicity | Master's degree or above | Married | Yes | G1 P0 | 20.96 | 25 |
| 3 | 32 | Han ethnicity | Master's degree or above | Married | No | G1 P0 | 23.51 | 27 |
| 4 | 32 | Han ethnicity | Master's degree or above | Married | Yes | G1 P0 | 18.82 | 28 |
| 5 | 36 | Han ethnicity | Master's degree or above | Married | No | G3 P1 | 17.69 | 24 |
| 6 | 34 | Han ethnicity | Bachelor's degree | Married | Yes | G1 P0 | 29.72 | 26 |
| 7 | 30 | Han ethnicity | Bachelor's degree | Married | No | G2 P0 | 24.01 | 30 |
| 8 | 36 | Han ethnicity | Bachelor's degree | Married | Yes | G1 P0 | 23.46 | 25 |
| 9 | 30 | Han ethnicity | Associate degree | Married | No | G3 P0 | 22.00 | 25 |
| 10 | 31 | Mongol ethnicity | Master's degree or above | Married | Yes | G2 P0 | 22.58 | 25 |
| 11 | 46 | Han ethnicity | Bachelor's degree | Married | No | G1 P0 | 20.93 | 26 |
| 12 | 30 | Han ethnicity | Master's degree or above | Married | No | G1 P0 | 19.84 | 24 |
| 13 | 34 | Han ethnicity | Master's degree or above | Married | No | G2 P0 | 22.72 | 27 |
| 14 | 31 | Han ethnicity | Master's degree or above | Married | Yes | G2 P0 | 25.73 | 25 |
| 15 | 30 | Manchu ethnicity | Bachelor's degree | Married | No | G2 P0 | 20.57 | 26 |
| 16 | 44 | Mongol ethnicity | Master's degree or above | Married | No | G5 P0 | 21.34 | 30 |
| 17 | 36 | Han ethnicity | Bachelor's degree | Married | No | G1 P0 | 21.09 | 26 |
| 18 | 29 | Han ethnicity | Bachelor's degree | Married | Yes | G1 P0 | 19.00 | 25 |
| 19 | 34 | Manchu ethnicity | Bachelor's degree | Married | No | G1 P0 | 28.13 | 24 |
| 20 | 33 | Han ethnicity | Associate degree | Married | No | G1 P0 | 33.30 | 25 |
| 21 | 39 | Han ethnicity | Master's degree or above | Married | Yes | G2 P0 | 21.22 | 28 |

Gestational diabetes mellitus, GDM; gravidity, G; parity, P; body mass index, BMI.
