# Supplementary material for: Developing a comprehensive structured program for managing gestational diabetes mellitus and preventing type 2 diabetes mellitus in Chinese women: a multi-method study
Source: Front Endocrinol (Lausanne). 2025 Aug 1;16:1627702. doi: 10.3389/fendo.2025.1627702 (PMC12353735; doi:10.3389/fendo.2025.1627702)
Supplement: Supplementary Figure 1 — PRISMA Flow Diagram. [file DataSheet1.zip › Table 7.DOCX]

**Supplementary Table 7** Needs and preferences of women with GDM in the interviews.

| **Theme** | **Category** | **Subcategory** | **Quote** |
| --- | --- | --- | --- |
| GDM-related knowledge | What is GDM | | ***P1****: "At the time, I didn't know what this disease was, mainly because I had never heard of it, so I was hoping the doctor could give me some guidance."*  ***P4****: "After my diagnosis, first of all, I hoped someone could explain to me what this condition is, what risks it poses, and what I should do about it. Among these, knowing what to do is the most important."* |
|  | Mechanisms and risk factors | | ***P2****: "My main concern is to first figure out the cause. Is it related to my diet? Or is it a family genetic issue? Or is it something that naturally occurs during pregnancy? Finding the cause would make me feel more at ease and help me find a solution."*  ***P14****: "Actually, I don't know much about gestational diabetes. I'm wondering, why does gestational diabetes even happen?"*  ***P19****: "I want to know what the symptoms are for pregnant women who are at high risk of gestational diabetes before pregnancy. Are there any symptoms of low blood* [*glucose*](javascript:;)*? Also, I had a hard time controlling my weight before pregnancy. Even though I ate very little, I would still gain weight. I'm not sure if this has anything to do with high blood glucose."* |
|  | The short- and long-term impacts on mothers and infants | | ***P12****: "After finding out yesterday that I have gestational diabetes, I had a lot of questions, but the doctor was very busy, so I didn't get a chance to ask in detail. I want to know if this will actually have any impact—on my own health, on my postpartum recovery, and on my baby. I’m hoping for a comprehensive answer, but so far, I haven't found anything that fully addresses my concerns."*  ***P13****: "I've done some research on the potential negative effects of gestational diabetes, and I’m wondering if the risks to myself or my baby are really as serious as they say."* |
|  | Postpartum blood glucose changes | | ***P14****: "After giving birth, is it possible that I might experience some high blood sugar issues, like type 2 diabetes? I would appreciate guidance and monitoring from the doctor."*  ***P19****: "I'm currently doing my best to control my blood glucose, but I want to know if it will return to normal levels after I give birth."* |
| Treatment of GDM | Dietary Guidance | Methods of food selection | ***P1****: "After the diagnosis, I'm not really sure how to choose the right foods because I have my own eating habits, and I've never really thought about these things before."*  ***P9****: "I want to know, if I stop eating fruit, will my blood glucose return to normal? Or, which fruits are healthier?"* |
|  |  | Assessment and guidance of intake | ***P3****: "In terms of diet, although I’m consciously trying to make adjustments, I’m not really clear on the specific recommended amounts, or which foods I’ve been eating too much of and which ones I’ve been eating too little of."*  ***P7****: "Although I’ve managed my sugar intake before, I probably used some very basic methods. I'm not sure about the specifics of how to do it or whether the amounts I'm eating are appropriate, so I might need some guidance in this area."* |
|  |  | Meal distribution methods | ***P12****: "I'm really busy with work. So, I hope I can get some guidance on snacks, considering my work schedule."*  ***P16****: "I often get really hungry after 11 or 12 at night, but it's already late, and I'm worried eating might be bad for my blood glucose. What's the best way to have a snack in this situation?"* |
|  |  | Individualized dietary guidance | ***P15****: "Diet should be based on our current pregnancy week and weight, and a suitable meal plan should be created. Then, we follow the meal plan when we eat."*  ***P17****: "After being diagnosed with gestational diabetes, my diet changed suddenly, and I'm having a hard time adjusting. As a result, I might need some recipes that are better suited for me."* |
|  |  | Understanding Sugar-Free Foods | ***P18****: "Besides basic dietary advice, I think it's also important to include knowledge about the* *Sugar-Free Foods. Before I started managing my blood glucose, I had no idea it would be so difficult. In the beginning, it's easy to fall into all sorts of traps, like foods labeled as sugar-free that actually have too many calories, or delicious breads that are basically just oil. I recommend the documentary That Sugar Film."* |
|  | Exercise guidance | | ***P6****: "I mainly want to learn about diet, exercise, and general lifestyle habits."*  ***P11****: "I want someone to tell me how to manage gestational diabetes, like giving guidance on diet and exercise."*  ***P15****: "Moderate exercise can help keep blood sugar stable, but pregnancy can come with various symptoms, like sciatica and pubic symphysis pain. How should I adjust my exercise routine if I experience these issues?"* |
|  | Guidance on the use of medicines | Indications for change of treatment | ***P17****: "Today, the doctor told me to control my blood sugar for two weeks, but I'm not sure how the situation will be handled if my blood glucose is either within or outside the target range after the two weeks."*  ***P8****: "I’ve heard that some pregnant women use insulin, so I’d like to know under what circumstances insulin would be added."* |
|  |  | How to use and preserve insulin | ***P16****: "I’m already using insulin, and I’d like to know how it should be stored. Right now, I’m not keeping it in the fridge."* |
| Glucose monitoring | Blood Glucose Monitoring and Instrument Use | | ***P5****: "Dietary guidelines definitely need to be provided, and specific instructions on how to monitor blood glucose, how to use a blood glucose meter, or other devices for monitoring blood* [*glucose*](javascript:;)*are also necessary."*  ***P8****: "I want to learn how to conduct regular check-ups and blood glucose monitoring to help me analyze and understand my blood glucose.”* |
|  | Diverse blood glucose monitoring methods | | ***P20****: "I'm a bit afraid of pricking my finger to test my blood glucose, and I hope there are multiple blood sugar monitoring methods that balance both accuracy and convenience."* |
| Guidelines for the prevention of postpartum T2DM | Postpartum follow-up | | ***P14****: "If my risk of developing T2DM in the future is indeed high, I also need to know how to monitor my blood glucose after childbirth, such as how often I should visit an endocrinologist."*  ***P5****: "I've read online that women with gestational diabetes are also at higher risk for blood sugar issues after childbirth, so I want to know what to watch out for post-delivery and how to specifically prevent these blood sugar problems."* |
|  | Guidance on T2DM prevention | | ***P5****: "I've read online that women with gestational diabetes are also at higher risk for blood sugar issues after childbirth, so I want to know what to watch out for post-delivery and how to specifically prevent these blood sugar problems."*  ***P14****: "After childbirth, I want to know if I might develop high blood glucose issues, like type 2 diabetes. Therefore, I need guidance and monitoring from my doctor."* |
|  | Importance of continued dietary management in the postpartum period | | ***P10****: "I want to know how it affects pregnancy and whether it will have any impact after childbirth. Do I still need to manage it postpartum?"*  ***P19****: "Since I've been controlling my blood sugar, my weight has been well-maintained, and I mainly focus on controlling my intake of staple foods, trying to eat more whole grains. However, after giving birth, I'm not sure if I need to continue managing it this way."* |
| Mental health support | Improvement of sleep quality | | ***P6****: "My lifestyle habits aren't very good; I often stay up late because I work the night shift, and my sleep quality isn't great. So, I'd like to know how I can improve my sleep."* |
|  | Alleviating GDM-related anxiety | | ***P11****: "I was a bit worried when I was first diagnosed, afraid there might be some negative effects. But if someone had given me guidance, I wouldn't have been so worried."*  ***P21****: "I know doctors are very busy and have to see a lot of patients every day. However, if doctors could pay a little more attention to patients' emotions, it might actually help with their recovery."* |
| Health education settings | Strategies for health education | Strategies to improve adherence to lifestyle management | ***P2****: "Everyone probably knows, to some extent, what is healthy to do after being diagnosed with gestational diabetes, but sometimes we're not willing to do it or just can't manage to follow through."*  ***P12****: "I hope there are ways to help improve my awareness of health management. Because if I'm not reminded, I tend to forget. Sometimes, when I get busy, I don't even have time to eat, let alone remember what I'm supposed to be paying attention to."* |
|  | Health education providers | Professionals, doctors, and experienced individuals (ranked from highest to lowest preference) | ***P15:*** *“I think anyone with relevant experience can do it.”*  ***P4:*** *“Definitely need a professional to guide me, but I don't really think about the other aspects that much.”*  ***P18:*** *“Regarding who will be lecturing, I think it's fine for a professional to do so.”*  ***P20:*** *“When it comes to who should provide health education, if I had to rank them, doctors at hospitals would definitely be the top choice, followed by scholarly literature, and then online resources like pregnancy blogs and notes.”* |
|  | Forms of health education | No preference for health education settings | ***P16****: " I don't have any particular idea of how to arrange it. It's all good.”*  ***P20****: "I don't have a special preference for the format of health education. I'm open to all kinds of information and will ultimately filter it myself. This might be related to the industries my family and I are in."*  ***P17****: "I don't have any special requirements."*  ***P18****: "I don't have a particular preference. I can accept both offline and online formats. Since my home is not far from the hospital, I mainly want to receive more meaningful information."* |
|  |  | Location: near the hospital, online | ***P12****: "As for the location of the health education, I think anywhere near this hospital would be fine."*  ***P15****: "Since I'm usually busy with work, online health education would be the best option for me."* |
|  |  | Time: weekends, on the day of the prenatal checkup | ***P12****: "I suggest the classes be held on weekends because I still work on weekdays during my pregnancy."*  ***P14****: "I prefer attending the class on the same day as my prenatal checkup, since my home is a bit far from the hospital, and it's a hassle to make a separate trip.”* |
|  |  | Provide practical and actionable health education materials | ***P12****: "After being diagnosed with GDM, the doctor asked me to take a picture of the dietary recommendations posted on the wall in the examination room. For this, I would prefer an electronic or paper version of the material instead of having to take a photo. It's not practical for me to constantly pull up photos on my phone, and flipping through pictures is inconvenient. Plus, the quality of my phone's camera isn't that great."* |
|  |  | Family participation | ***P12****: "In addition to paying attention myself, the person who prepares my meals really needs to pay more attention. I wish there were materials I could share with my family to raise their awareness. When my family is preparing food for their daughter, daughter-in-law, or partner, they should be mindful of the dietary precautions for their daughter, daughter-in-law, or partner."*  ***P16****: "My husband is overweight, and his eating habits are really bad. He cooks with too much oil and salt. So, when I'm at home, it's hard for me to find the right kind of food to eat. I really hope he can get some education on this as well."*  ***P18****: “Family support is more important than I expected. In the beginning, when I tried to adjust my diet on my own, it always ended in failure. I would often think, "I'll just make do with this meal" when I was tired, and after a few meals like that, my blood glucose would get out of control, making me want to give up. But once my family started helping, I had external supervision, which made a huge difference. It’s much better than doing it alone — at least when I overeat, someone is there to remind me. Additionally, family members need to have a certain level of health awareness. If they aren’t able to provide proper supervision, it’s easy to slip back into bad habits. That’s why I hope to bring my family along to the classes.”* |
|  |  | Group-based | ***P12****: “I prefer group sessions. That way, I can interact with the instructor or other pregnant women.”* |

[Gestational](javascript:;) [diabetes](javascript:;) [mellitus](javascript:;), GDM; type 2 diabetes mellitus, T2DM.
