# Supplementary figures and images for: Developing a comprehensive structured program for managing gestational diabetes mellitus and preventing type 2 diabetes mellitus in Chinese women: a multi-method study
Source: Front Endocrinol (Lausanne). 2025 Aug 1;16:1627702. doi: 10.3389/fendo.2025.1627702 (PMC12353735; doi:10.3389/fendo.2025.1627702)

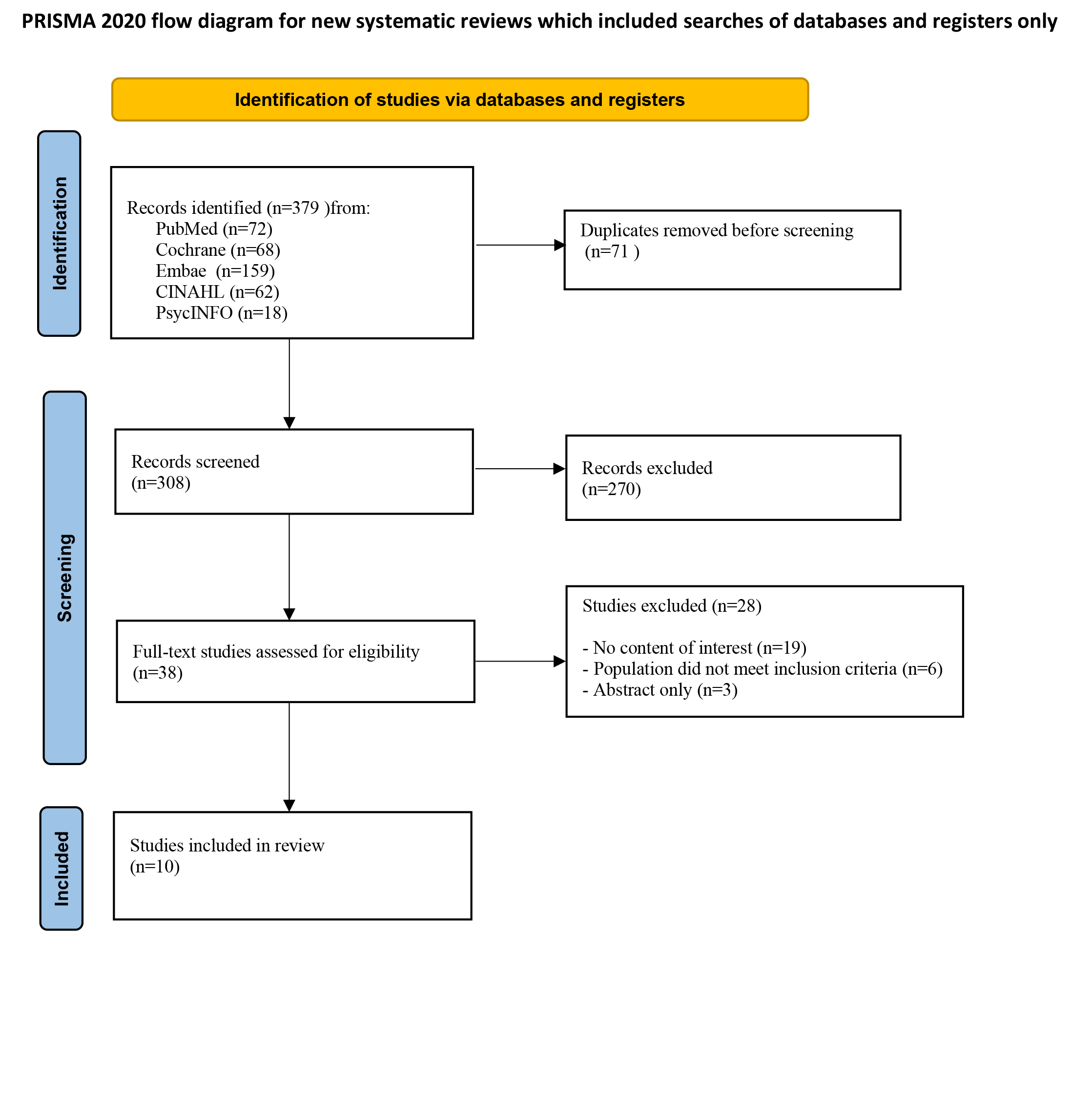

Supplement: Supplementary Figure 1 — PRISMA Flow Diagram. [file DataSheet1.zip › Image 1.TIF]

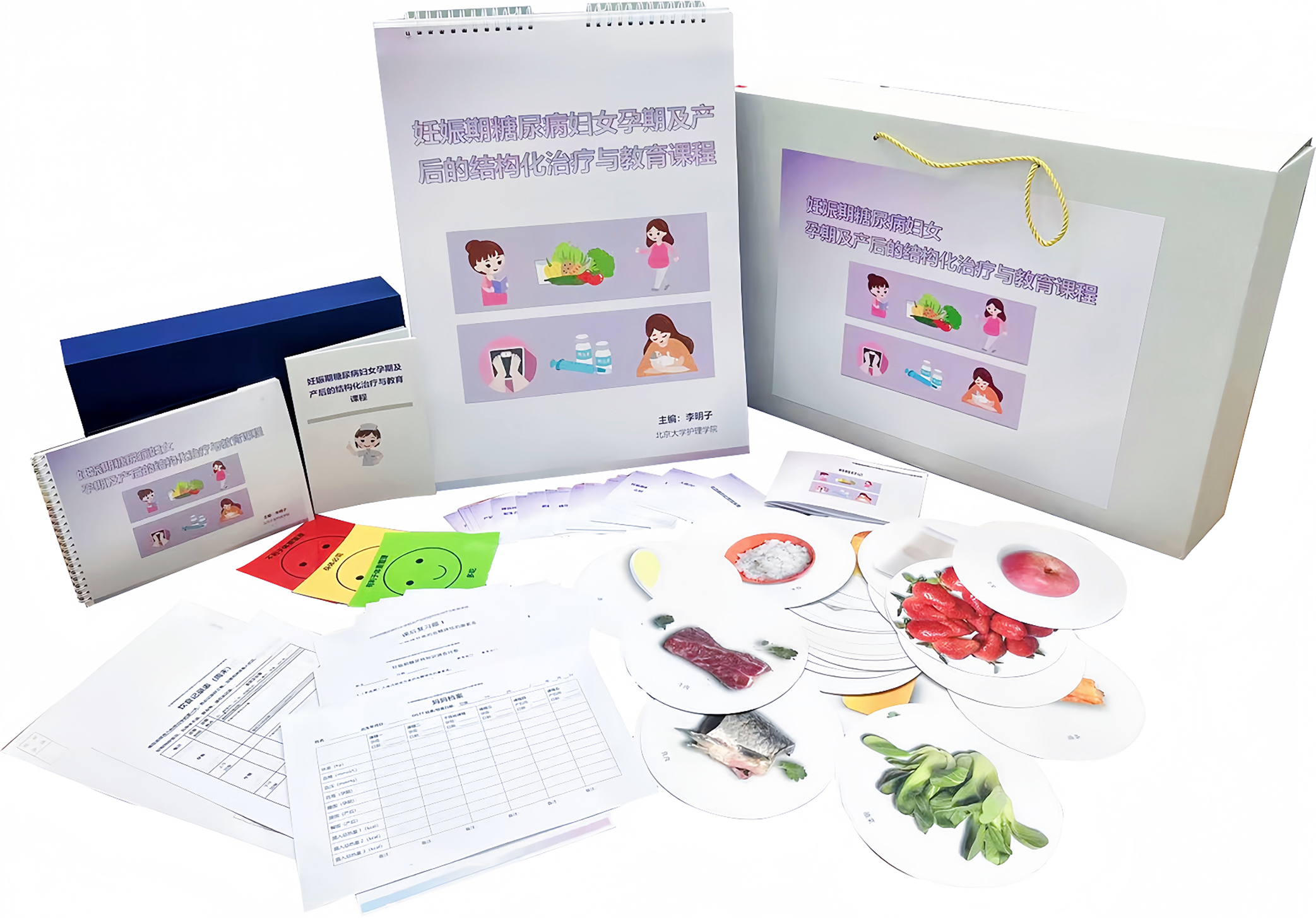

Supplement: Supplementary Figure 1 — PRISMA Flow Diagram. [file DataSheet1.zip › Image 2.TIF]
